# A single-cell resolved cell-cell communication model explains lineage commitment in hematopoiesis

**DOI:** 10.1242/dev.199779

**Published:** 2021-12-22

**Authors:** Megan K. Rommelfanger, Adam L. MacLean

**Affiliations:** Department of Quantitative and Computational Biology, University of Southern California, 1050 Childs Way, Los Angeles, CA 90089, USA

**Keywords:** Cell fate, Hematopoiesis, Mathematical modeling, Gene regulatory network, Bistability

## Abstract

Cells do not make fate decisions independently. Arguably, every cell-fate decision occurs in response to environmental signals. In many cases, cell-cell communication alters the dynamics of the internal gene regulatory network of a cell to initiate cell-fate transitions, yet models rarely take this into account. Here, we have developed a multiscale perspective to study the granulocyte-monocyte versus megakaryocyte-erythrocyte fate decisions. This transition is dictated by the GATA1-PU.1 network: a classical example of a bistable cell-fate system. We show that, for a wide range of cell communication topologies, even subtle changes in signaling can have pronounced effects on cell-fate decisions. We go on to show how cell-cell coupling through signaling can spontaneously break the symmetry of a homogenous cell population. Noise, both intrinsic and extrinsic, shapes the decision landscape profoundly, and affects the transcriptional dynamics underlying this important hematopoietic cell-fate decision-making system.

This article has an associated ‘The people behind the papers’ interview.

## Abstract (precis)

**Summary:** Through theory and computational modeling, cell-cell communication is revealed to be a crucial and under-appreciated determinant of cell-fate decision-making during hematopoiesis.

## INTRODUCTION

The production of mature cell types from stem cells and progenitors is essential for development and organ homeostasis. Nevertheless, in few cases, are we able to fully specify the conditions necessary to drive stem cell differentiation towards a particular cell lineage. Stem cell differentiation is controlled by cell-internal and -external signals that, in turn, control the transcriptional state of the cell and specify its eventual fate (Roeder, 2006; Laurenti and Göttgens, 2018; Graf and Enver, 2009). These changes in transcriptional state are often irreversible and involve binary choices. Thus, multipotent cells, through successive binary lineage specification choices, eventually become committed to a specific lineage and cell-fate. Significant unanswered questions remain regarding the cell-fate decisions that dictate lineage specification of stem/progenitor cells: How large is the role of extracellular signaling in regulating cell differentiation? What mechanisms allow cells from initially homogeneous or clonal populations to converge to different lineages? How does one population of progenitor cells maintain stable heterogeneous subpopulations of committed cell types?

During lineage specification, changes in gene expression are controlled by gene regulatory networks (GRNs), consisting of genes and their protein products (transcription factors), which are able to regulate the expression of various genes, including other transcription factors, creating feedback loops (Tyson et al., 2003). Codified through mathematical models, GRNs can be studied in light of their steady states, where each steady state can represent a committed cell fate. Certain GRN topologies permit bistability, i.e. more than one steady state can be reached for a single set of biological conditions (Craciun et al., 2006): such topologies are frequently observed in networks that control cell-fate decisions (Alon, 2007; Kamenz et al., 2021). The GRN topology that dictates a particular lineage decision is generally conserved across cells, thereby providing insight into the intracellular dynamics that occur during cell differentiation. Crucially, the GRNs that instigate or reinforce lineage decisions are not only controlled by cell-intrinsic networks, but also by cell-extrinsic signals.

The GRN topology considered in this work consists of two mutually repressive genes, a topology frequently observed among gene networks mediating lineage decisions (Pal et al., 2014; Ferrell, 2002). One such lineage decision occurs during hematopoiesis: myeloid progenitor cells make a binary choice between commitment to the megakaryocyte-erythroid (ME) lineage or the granulocyte-monocyte (GM) lineage. *GATA1* and *PU.1* (*SPI1*) mutually inhibit one another; *GATA1* is expressed in the ME lineage and *PU.1* is expressed in the GM lineage. This mutually repressive GRN has been extensively studied and characterized in models, mostly consisting of ordinary differential equations that permit bistability, thus enabling investigation into the dynamics of this myeloid lineage decision (Chickarmane et al., 2009; Duff et al., 2012; Huang et al., 2007; Chang et al., 2008; Roeder and Glauche, 2006).

Given the *GATA1-PU.1* mutual inhibitory loop that leads to bistability, changing the initial conditions (gene expression levels) within a bistable region is sufficient to change the cell fate. It has thus been proposed that random fluctuations of *GATA1* and *PU.1* levels are primarily responsible for determining cell fate in the bipotent progenitor population that has dual ME and GM lineage potential (Chang et al., 2008). More recently, this notion was challenged by a study that used a double reporter mouse (PU.1^eGFP^GATA1^mCherry^) to show that that random fluctuations of *PU.1* and *GATA1* (*Spi1* and *Gata1* in mouse) are insufficient to initiate the cell-fate decision between ME and GM lineages (Hoppe et al., 2016). Hoppe et al. also provide strong evidence that ME versus GM lineage specification cannot be determined solely from initial ratios of *PU.1* to *GATA1* expression.

As suggested by Hoppe et al. (Hoppe et al., 2016), extracellular signaling could resolve this controversy. Cell signaling pathways, including Jak/Stat, Wnt/β-catenin, MAPK/ERK/p38 and PI3K/Akt have been recently identified as being crucially important during GM and ME cell-fate specification (Wang et al., 2021). Previous studies of hematopoiesis both *in vivo* and *in vitro* have also hinted that cell-cell signaling influences cell-fate decision-making. *In vivo*, it has been shown that the number of hematopoietic cells transplanted affects the phenotype of the regenerated blood system in the recipient (Brewer et al., 2016). *In vitro*, hematopoietic stem and progenitor cells placed into wells of different cavity sizes, and therefore with different numbers of neighboring cells, gave rise to different cell-fate patterns (Müller et al., 2015). These two lines of evidence suggest that cell-cell signaling plays a role in hematopoietic cell-fate specification. Both the theoretical gap and the experimental data thus point to a pressing need to consider the impact of extracellular signaling dynamics when modeling hematopoietic differentiation.

The role of extracellular signaling, however, has been chronically understudied in gene regulatory network models. Extracellular signaling through paracrine factors enables populations of cells to share information and coordinate behaviors over short distances, and is implicated in a range of cell-fate behaviors from developmental branching (Lambert et al., 2018; Iber and Menshykau, 2013) to patterning (Scholes et al., 2019) and migration (McLennan et al., 2015). A seemingly simple question motivated this work: how do intercellular signaling networks impact myeloid lineage specification, as controlled by the *GATA1-PU.1* gene regulatory network?

Several studies have characterized the impact on cell fate of cell-cell communication through extracellular signals. One approach taken is to model the internal gene dynamics with differential equations and model cell-cell communication by allowing for molecular diffusion of proteins between neighboring cells (Ellison et al., 2016; Mugler et al., 2016; Smith and Grima, 2018). These methods must neglect anisotropic effects, and assume that signaling between cells occurs on the same timescale as intracellular dynamics; yet the processes can occur on vastly different timescales. Other approaches have relaxed this assumption, permitting different timescales by modeling the response time of a cell to a signal received as a random variable (Thurley et al., 2018). This approach, however, omits any detail of the intracellular dynamics, which are often crucial to the response; GRNs are the enforcing mechanisms of the phenotypic switches. In order to model the individual behaviors of a population of communicating cells, the gap between intracellular dynamics and extracellular signaling must be bridged. A model of cell-fate decision-making is needed that permits cell-cell communication while allowing for both description of the cell-internal dynamics and of the extracellular signaling dynamics without making diffusion-like approximations.

We present a multiscale model that bridges this gap. We assume deterministic dynamics within each cell and thus model the cell-internal GRN with ordinary differential equations (ODEs). We assume that signals sent between cells can be described by a Poisson process. Signals received by cells alter the internal GRN dynamics through their effects on parameters of the ODE model. We test this model in a large range of different intercellular signaling topologies. We find that the addition of cell-cell communication to GRN dynamics leads to model outcomes (cell-fate distributions) becoming probabilistic: cell-fate choice probabilities now depend on the position of the cell in a particular signaling topology. We discover that the model can intuitively characterize cell-cell coupling, changes to which impact the cell-fate decisions made, which can lead to mixtures of heterogeneous cell types within a population. We also study how noise impacts fate outcomes: we find that although both intrinsic and extrinsic noise alter the cell-fate decision-making boundaries, extrinsic noise is the dominant driver of cell-fate variability. Finally, we study how cell fates are influenced by spatial architecture, and we demonstrate that the extent to which cell fates are coupled is controlled by proximity in spatially structured cell-cell signaling systems.

## RESULTS

### Cell-cell communication over a wide range of signaling topologies leads to divergent cell fates

**Fig. 1.**
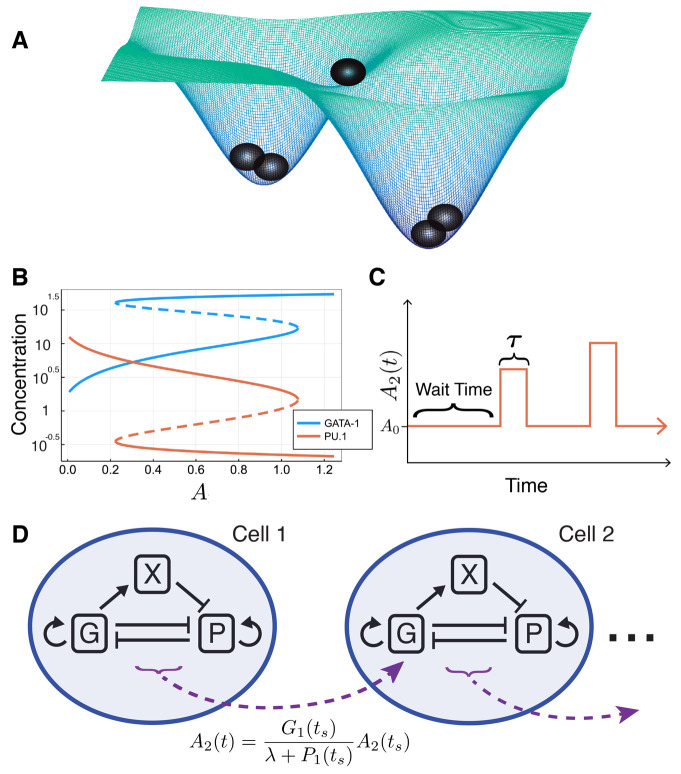
**Overview of multiscale cell-fate commitment model.** (A) Illustration of cells in a two-attractor state model (such as the GATA1-PU.1 inhibitory loop). (B) Bifurcation diagram for the system of ODEs described by Eqns 1-3, with respect to the external signaling parameter *A*. (C) Cell-cell communication via signals sent between cells (e.g. from cell 1 to cell 2) is modeled by a Poisson process, with wait times sampled from an exponential distribution and fixed signaling ‘pulses’ of length ***τ***, where the value of *A*_**2**_ is set by Eqns 4 or 5. (D) Full model schematic, depicting the cell-internal gene regulatory network of {G,P,X} modeled by ODEs and the external signal *A*_2_ modeled by the Poisson process in C.

To determine how cell-cell communication instructs hematopoietic lineage specification, we construct a multiscale model of the bifurcation point in hematopoiesis that separates erythroid/megakaryocyte from granulocyte/monocyte lineages (Fig. 1A). This lineage decision is controlled in part by the mutually antagonistic genes *GATA1* (*G*) and *PU.1* (*P*), giving rise to a bistable model (Fig. 1B). We model the cell-internal GRN coupled to a cell-cell communication motif that can alter the cell-internal expression of *G* in neighboring cells, according to a Poisson process (Fig. 1C,D). Full details of the model specification can be found in the Materials and Methods.

We test the model for a wide range of signaling topologies. We sought to ensure that core assumptions were upheld, namely that all cells eventually reach a steady state, even in the presence of noisy signaling, and that cells exhibit bistability with respect to *A*_0_ (the initial value of *A*). These behaviors were preserved in all signaling topologies tested. Moreover, we found that the addition of cell-cell signaling to a previously deterministic model results in probabilistic cell-fate choices.

**Fig. 2.**
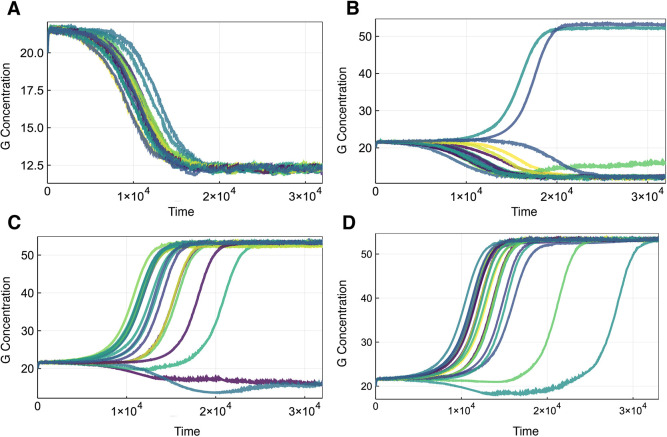
**Sample trajectories of *GATA1* (G) for cells signaling in a loop.** Representative simulations of a 20-cell loop signaling topology. Colors used only to distinguish the trajectories. Across all trajectories, the signaling parameter is set to ***λ***=28. (A) *A*_0_=0.995. (B) *A*_0_=0.9955. (C,D) *A*_0_=1.0.

**Fig. 3.**
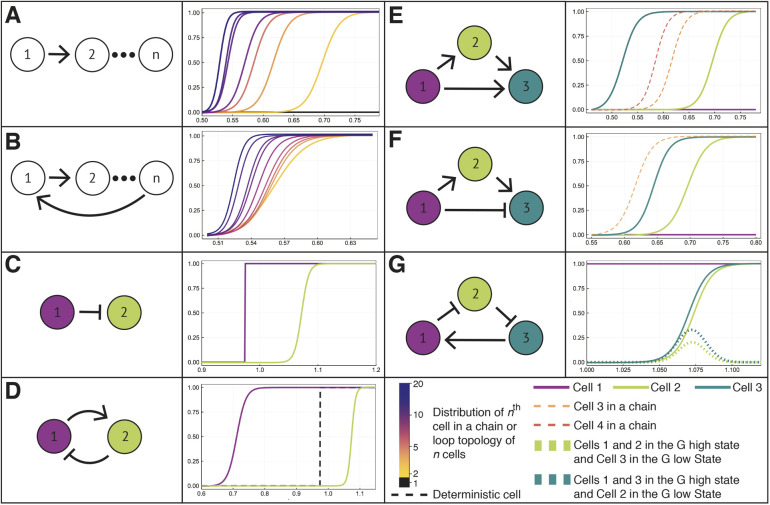
**Cell-fate commitment across a range of signaling topologies.** Schematic of different cell signaling topologies (left) and the corresponding probability distributions: the probability that each cell in the network will commit to the G high steady-state, *P*(*G* high), for different values of *A*_0_. (A) Chains of cells of length *n*. Each probability distribution corresponds to one cell in the chain. (B) Loops of cells of size *n*. In a loop of fixed size, each cell from 2 to *n* has the same probability distribution; thus, one curve is plotted for each loop of *n* cells. (C) A chain of two cells with a dissensus signal: the distribution of fates of cell 2 is shifted to the right, i.e. it commits at higher values, compared with consensus signal in A, where the distribution of cell 2 is shifted to the left. (D) A loop of two cells with one consensus and one dissensus signal; dashed line indicates the distribution of a single cell that receives no signals (deterministic). We cannot observe the fate where cell 1 is in the G low state and cell 2 is in the G high state. (E) Three-cell feed-forward motif; cells 3 (dashed orange) and 4 (dashed red) from a chain of cells marked for comparison. We see that receiving multiple signals results in a non-additive synergistic effect. (F) Feed-forward motif with a dissensus signal; cell 3 (dashed orange) from a chain of cells marked for comparison. (G) Loop of three cells with two dissensus signals. Dashed lines indicate the probabilities that: (1) cells 1 and 2 are in G high state, while cell 3 is in G low state (dark green); or (2) cells 1 and 3 are in G high state, whereas cell 2 is in the G low state (light green).

In Fig. 2, simulated trajectories for a 20-cell loop topology (see also Fig. 3B) are shown. We plot only the concentration of *G* as a proxy for fate choice, as its state is sufficient to determine the steady state of the full system (if *G* is high at steady state, *P* is low, and vice versa); as illustrated by the trajectories of both *G* and *P* (Fig. S1). These trajectories illustrate the variety of cell-fate distributions that are observed in the presence of cell-cell communication (fate convergence to either the high or low state, and fate divergence). In Fig. 2C,D, the values of *A*_0_ are equal, demonstrating that *A*_0_ does not determine the proportion of cells in each state. Rather, for each value of *A*_0_, the probability of each cell converging to a certain state can be computed.

In analyzing how signals propagate down chains of cells, two striking observations are made. First, each additional signal shifts the *P*(*G* high) curve (the probability of reaching the *G* high state) to the left, although these shifts are successively smaller down the chain such that eventually the distributions converge. Second, a ‘sharpening’ of the *P*(*G* high) distributions occurs whereby the region of uncertain fate decreases for cells further down a chain (Fig. 3A). This is a hallmark of cooperativity: accumulated signals by cell-cell communication act to reinforce cell-fate decision-making, leading to regions of uncertainty that are decreasing with the total number of signals that are acting on a cell.

In loops, all cells converge to the same fate for each simulated trajectory, therefore every cell in the model has the same probability distribution (below we will see that the coordination of fates in all cells within a loop signaling topology can be broken by changing the cell coupling strength). The probability distribution for a cell loop depends on the number of cells in the loop, and we see that for loops of size *n*, the behaviors observed above for cell chains are recapitulated (Fig. 3B). For both cell loops and chains, we observe that, as the number of cells in the signaling topology increases, so does cell response fidelity, i.e. sharpness of the curve (Fig. 3A,B), a prediction that matches experimental data on paracrine signaling in wound healing (Handly et al., 2015). This holds true for large topologies up to at least 100 cells in size (Fig. S2). Moreover, the limiting distributions for the cell chain and the cell loop are the same, highlighting an important underlying convergence.

If we consider a dissensus signal (the opposite of a consensus signal), the fate probability distribution of cell 2 shifts in the opposite direction than previously (Fig. 3C), relative to the value of *A*_0_, at which cell 1 switches lineages. When an inhibition signal is incorporated into a loop (Fig. 3D), the two cells in the loop no longer coordinate lineages for every trajectory as they did when all signals were regular. Notably, in Fig. 3D there exists a large range of *A*_0_ for which cell 1 converges to the *G* high state and cell 2 converges to the *G* low state, i.e. dissensus signals significantly change cell-fate behaviors in both chains and loops of cells.

In a feed-forward signaling motif (Fig. 3E), multiple promoting signals reinforce the lineage choice of cell 3, thus increasing cooperativity and as a result the cell commits to the *G* high state at smaller values of *A*_0_ than it would in a cell chain. Other three-cell signaling topologies also display interesting behaviors (Fig. 3F,G and Fig. S3). In an incoherent feed-forward motif, we observe a multiplicative effect between the dissensus signal from cell 1 and the consensus signal from cell 1 via cell 2 (Fig. 3F). The addition of the dissensus signal results in the distribution of cell 3 being shifted to the right relative to a corresponding cell in a chain. For a doubly dissensus topology (Fig. 3G), we find that for a region of *A*_0_ the cell fates can diverge (two cells in the high state; the third in the low state) with non-zero probability.

Consensus or dissensus signaling can occur when the signaling pathway mediating the signal contains a positive- or a negative-feedback loop, respectively. There are many such recorded examples of signaling in hematopoiesis and other contexts. One example of a consensus signal directly implicated in the GM versus ME cell-fate choice is mediated by the cytokine TNFα, which activates the p38 MAPK pathway, in turn activating TNFα as a target gene (Zarubin and Han, 2005; Wang et al., 2021). A principal example of dissensus signaling, both in the context of hematopoiesis and other cellular processes, is the Delta-Notch signaling pathway (Ohishi et al., 2003), which leads to consecutively divergent patterns of activity.

We expanded our analysis of cell-cell communication to consider other extracellular signals, and their effects alone or in competition with each other. We studied a model of consensus signaling that impacts the activation of *PU.1* rather than *GATA1*, i.e. changes the parameter *B*. Cell-cell communication in this model impacts cell fate similarly to in the original, i.e. for a bistable region with respect to *B*_0_, lineage specification becomes probabilistic for simple cell signaling topologies (Fig. S4). We also studied a model with two competing extracellular signals, i.e. we changed both parameters *A* and *B* through independent Poisson processes. In the context of a two cell chain, we found that the joint influence of the signals sharpens the cell-fate distribution. Full characterization of the two-signals model has yet to be conducted as the size of the parameter space doubles, leading to challenges in fully elucidating the region of bistability.

The signaling topologies studied here pertain to small and idealized cell signaling networks. Nonetheless, their behaviors point to general and important effects of cell-cell communication. In all of the above experiments, the addition of cell-cell signaling transformed a population of homogeneous, independent and deterministic cells into one of heterogeneous cells that choose fates non-deterministically. Thus, given cells with identical initial conditions (i.e. transcriptional states), external signaling changes cell-fate outcomes at the population level. This corroborates a major finding of Hoppe et al. (Hoppe et al., 2016): that GMP/MEP cell-fate decisions are not predictable from initial transcription factor ratios alone. Our model not only supports this result, but offers an explanation. The missing determinant of cell-fate commitment – which acts to break the symmetry of an initially homogenous population of cells – is cell-cell communication.

### Varying the strength of cell-cell coupling results in different ratios of stable subpopulations of heterogeneous cell types

Through investigation of cell-cell communication in the multiscale model introduced above, we discovered that cell-fate divergence can occur under the control of the model parameter *λ*. We thus characterize *λ* as a ‘cell-cell decoupling’ mechanism. Analysis of cell-cell coupling and decoupling led to two significant findings: cell-cell communication can explain how a population of cells maintains stable subpopulations of heterogeneous cell types; and the distribution of cells that converge to each fate depends both on cell-cell coupling and on the external environment.

**Fig. 4.**
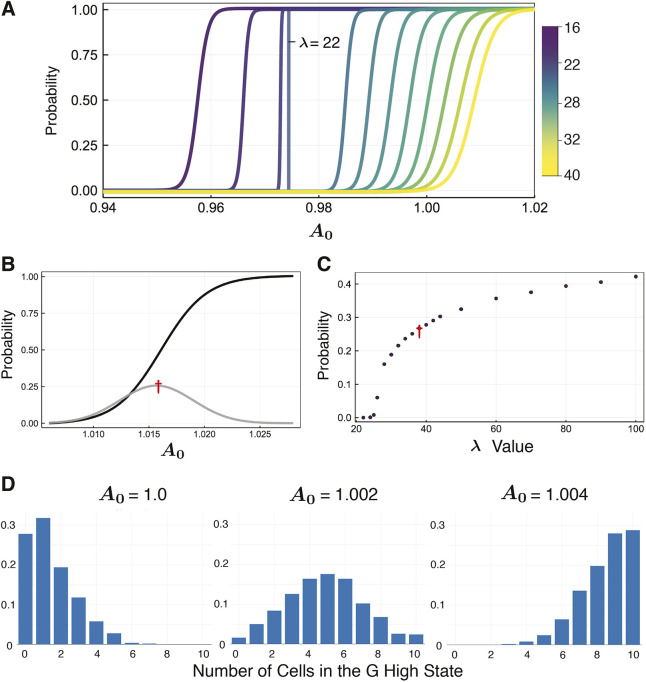
**Cell-fate coupling is controlled by the external signaling parameter.** (A) Probability distributions of cell-fate commitment to G high state for cell 2 in a chain of two cells for ***λ***∈[16, 40]. (B) Probability distributions for a loop of two cells as a function of *A*_0_, with ***λ***=38. Probability of cell-fate commitment to G high state (black); probability that the two cells will not commit to the same fate (gray), with the maximum of the distribution marked (red dagger). (C) For a loop of two cells, the maximum probability that the cells do not converge to the same lineage as ***λ*** varies; red dagger equivalent to that in B is marked for comparison. (D) Comparison of distributions for the number of cells that commit to the G high state in a loop of 10 cells with ***λ***=30.

Analysis of the effects of *λ* on consensus signaling topologies shows that – for a chain of two cells – as *λ* increases, the probability distribution of the second cell, *P*(*G* high), shifts to the right (Fig. 4A). Opposite results are observed when we analyzed the effects of *κ* on dissensus signaling topologies, where the probability distribution of the second cell shifts to the left as *κ* increases (Fig. S5). We identified *λ*=22 as close to a critical value, where cell-fate decision-making becomes switch-like. The value of *A*_0_ where the curve for *λ*=22 switches from one lineage to another is extremely close to the value at which a deterministic cell with the same initial conditions switches. Overall, *λ* determines the range over which the probability curve for the second cell is non-zero.

We next assessed how different values of *λ* changed coordination of cell-fate decisions between lineages. Previously, we observed that for any trajectory of a loop topology of any size, where *λ*=1, all cells converged to the same state, regardless of the value of *A*_0_. Furthermore, although all cells in the topology converge to the same lineage, the probability that they all converge to a given lineage depended on *A*_0_. To see whether or not this behavior was conserved for other values of *λ*, we tested a two cell loop topology with a range of *λ* values. We observed that there exists a value, λ*≈22, such that, for *λ*<λ*, all the cells coordinate their lineage just as we had seen before. For *λ*>λ*, the two cells do not always coordinate their lineage decisions. For example, Fig. S6 gives sample trajectories showing two cells in a loop converging to each combination of states. Fig. 4B shows the two cell loop results for *λ*=38, giving the probabilities that each cell converges to the *G* high state as well as the probabilities that the cells are decoupled, converging to opposite lineages. For each *λ*, we recorded the maximum value of the probability that the cells converge to different lineages. Fig. 4C shows how the maximum probability of cells converging to different lineages increases with *λ*.

Next, we looked at larger loops of 10 cells. For each value of *A*_0_, we simulated 500 trajectories and recorded how many cells were in the *G* high state verses the *G* low state. Fig. 4D shows results for *λ*=30, and *A*_0_= 1.0, 1.002 and 1.004. We see that the distribution of cells converging to each state changes with the value of *A*_0_. Further analysis of distributions with different values of *λ* can be found in Fig. S7.

We have identified an explicit mechanism by which cell-cell communication can break the symmetry of a homogeneous population of progenitor cells, and give rise to stable, heterogeneous populations of lineage-committed cells. The proportion of cells committed to each lineage depends on the external environment, *A*_0_, and the strength of cell-cell coupling due to signaling, *λ*. Moreover, these results show that fluctuations in the external environment can lead to shifts in the relative abundances of committed cell types. These results corroborate previous work that studied the generation of heterogeneous cell populations through stem cell differentiation (Mojtahedi et al., 2016), and showed that external signals (e.g. through cell-cell communication) are required both to maintain heterogeneous cell populations and to shift relative cell types abundances in response to environmental perturbations.

### Intrinsic and extrinsic noise alter cell-fate decision-making boundaries

**Fig. 5.**
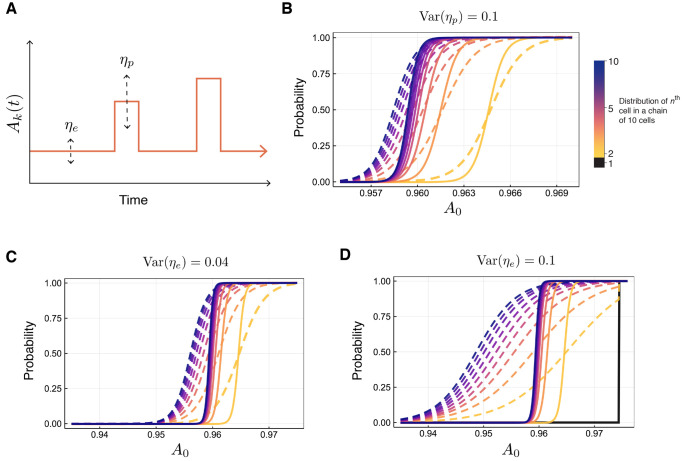
**Extrinsic and intrinsic sources of noise reduce fidelity of cell-fate choice.** (A) Schematic depicting how extrinsic noise (*η*_***e***_) and intrinsic noise (*η*_***p***_) are modeled through their effects on the signaling strength *A*_*k*_. (B) Probability distributions of cell-fate commitment (to G high state) for each cell in a ten-cell chain, with ***λ***=18: no added noise (solid lines); and with added noise (dashed lines). The colors darken as the position of the cell along the chain increases. The type of noise modeled is intrinsic, with Var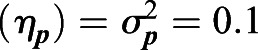. (C) As for B, but for extrinsic noise modeled, with Var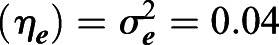. (D) As for B, but for extrinsic noise modeled, with Var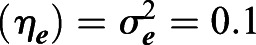.

We have until now assumed that signals are passed between cells with perfect fidelity. In fact, multiple sources of noise contribute to imperfect communication between cells, and the modeling framework here lends itself well to the investigation of the effects of intrinsic versus extrinsic noise (Hilfinger and Paulsson, 2011; Swain et al., 2002). We investigated the impact of two different sources of noise in cell-cell communication: due to cell-extrinsic factors, i.e. noise with respect to the extracellular environment (Eqn 7); or due to cell-intrinsic factors, i.e. noise due to signal transduction downstream of a paracrine signaling factor (Eqn 8). These sources of noise are represented in the model by varying either the baseline level of cell-cell communication (extrinsic noise) or the cell signaling pulse level, i.e. the intrinsic signal transduction noise (Fig. 5A).

For simple topologies, as we would expect, as the variance of either the extrinsic or the intrinsic noise increases, the observed variability in cell fate outcomes also increases (Fig. S8). This result also holds for larger cell-cell communication topologies, e.g. a ten-cell loop: intrinsic (*η*_*p*_) and extrinsic (*η*_*e*_) noise (Fig. 5B-D) both reduce the sensitivity of the cell-fate decision-making boundary. We also observe unexpected and striking results. First, not only are the probability distributions flattened by either noise source, but they are shifted to the left, i.e. intrinsic and extrinsic noise directly affect the decision-making boundary (by shifting its mean), as well as the sensitivity of cell-fate decision-making. Second, even under the presence of noise, we still observe the effects of cooperativity at play through the sharpening of distributions down a chain of cells (Fig. 5D). That is, individual noisy signals increase the variability (reduce the sensitivity) of cell-fate decision-making, but the cumulative effects of noisy signaling can at least partially compensate for this, and decrease the variability in cell-fate decision-making.

Through comparison of the relative effects of the intrinsic signal transduction noise (Fig. 5B) and the extrinsic extracellular noise (Fig. 5C,D), we see that the impact on the cell-fate decision-making boundary is much larger for extrinsic rather than intrinsic noise contributions. From inspection of Eqns 6 and 7, this is in part due to smaller duration of the pulse *τ* relative to the mean wait time *μ*. The result of which is that when 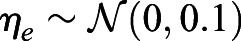 (Fig. 5D), the probability curves for cells 2-10 in the chain flatten to the extent that the cell fates are indeterminate regardless of position along the chain. Moreover, the probability curves for cells ≥2 along the chain intersect with the point at which cell one (which is deterministic) switches fates from the low to the high state (black line in Fig. 5D), thus forcing all other cells also into the high state with probability = 1. This coordination of cell fates is influenced by cell-cell coupling [here, we use *λ*=18<λ*, i.e. a value of *λ* for which we observe complete coupling (fate coordination) between cells]. The dominant impact of extrinsic over intrinsic noise is in agreement with previous works, including a study that quantified the contributions of extrinsic and intrinsic noise in the MAPK signaling pathway, and showed that extrinsic noise is the dominant driver of cell-to-cell variability (Filippi et al., 2016). It has also been shown that explicit extrinsic noise contributions are necessary to explain mRNA abundance distributions (Ham et al., 2020).

In summary, the effects of intrinsic and extrinsic noise on cell-cell communication topologies are to increase the variance of the resulting cell-fate distributions and (surprisingly) to alter the mean values of these probability distributions. In other words, the presence of noise alone can force cells to change lineages. The observed increases in the variability of cell-fate decision-making are maintained for large cell-cell communication topologies. A similar result was described in a study of cell-fate decision-making during early mouse gastrulation (Mohammed et al., 2017): transcriptional noise is greatest at the point of cell-fate decision-making (when epiblasts begin to differentiate). Our results reiterate the same point made by Mohammed et al.: that gene expression noise is beneficial during windows of cell-fate decision-making as it leads to an increased possible repertoire of cell fates. Our findings go further in that they suggest a rationale: that the increase in transcriptional noise results from noisy extracellular factors influencing cell-cell signaling during differentiation.

### Spatial structure in communication networks impacts cell-fate outcomes

**Fig. 6.**
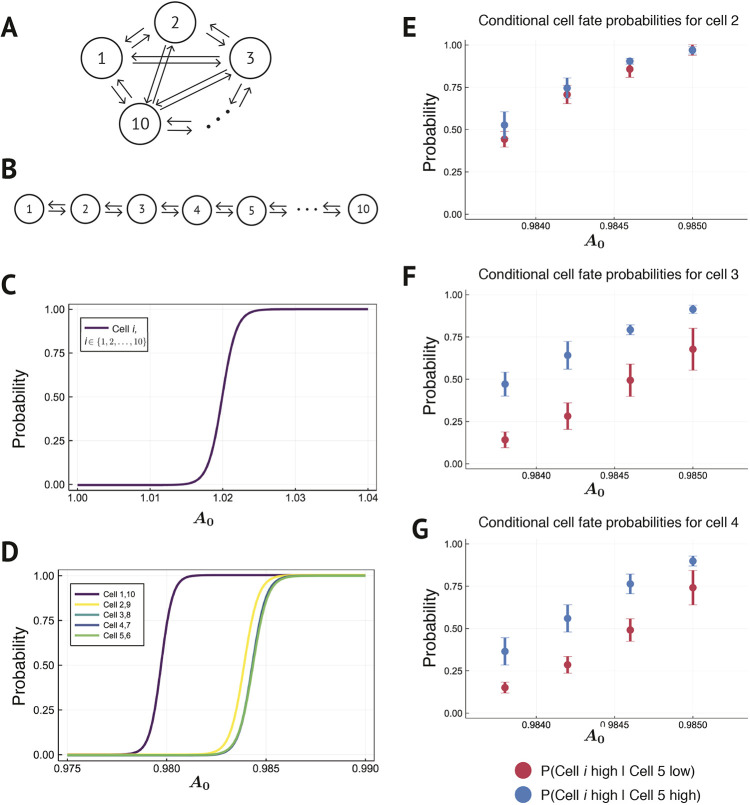
**Spatial organization of cells regulates patterns of cell-fate commitment.** Schematic representation of models for (A) a fully connected signaling topology and (B) a bidirectional chain signaling topology. (C,D) Probability distributions of the probability that each cell in the bidirectional chain (C) and fully connected (D) signaling topologies will commit to the G high steady state for different values of *A*_0_. (E-G) Probabilities that: (E) cell 2, (F) cell 3 or (G) cell 4 will commit to the G high steady state, conditional on the fate of cell 5. Cell 5 committing to the G high state (blue); cell 5 committing to the G low state (red). The model was simulated in *n*=10 independent experiments each with 100 trajectories. The mean of all 1000 simulations is given, and the error bars indicate the s.d. observed across the 10 samples.

Years of thorough investigation into the spatial organization of the bone marrow have revealed complex environmental interactions and stem cell niches (Lo Celso and Scadden, 2011; Morrison and Scadden, 2014; Baryawno et al., 2019; Upadhaya et al., 2020;Christodoulou et al., 2020); however, much less focus has been given to the locations of progenitor cells, including multipotent cells of GM and ME lineage potential. Moreover, the freedom of cell movement relative to, for example, epithelial tissues limit our ability to characterize spatial cell locations. Coupled with the current limitations on detecting cell-cell communication directly from data, it is not yet possible to infer spatial cell-cell communication networks in hematopoietic cells from data. Thus, to investigate the impact of spatial organization on cell fate, we investigated two topologies that characterize spatial extrema: a fully connected topology and a bidirectional chain of cells (Fig. 6A,B). These models use the ‘connectedness’ of a cell to characterize environments either lacking in spatial structure (fully connected topology) or highly structured (bidirectional cell chain).

We simulate ten-cell models for both the well-mixed and spatially restricted topologies, with the same model parameters and initial conditions. We used *λ*=28 and reduced the signaling pulse period to 1.0, to better distinguish the signals due to the large total number of signals being sent and received in the fully connected topology. In the fully connected model, each cell is topologically equivalent and has the same probability distribution of converging to the GM lineage (G high) state (Fig. 6C). However, in the spatially restricted bidirectional chain, the fate choice probability distribution is position dependent: the farther from the center of the chain, the more the distributions diverge (Fig. 6D). The spatial organization encapsulated in the bidirectional chain impacts hematopoietic lineage specification, even in a population of homogeneous cells.

To explore the spatial patterns of cell fate that emerge, we computed the probability of the commitment of an individual cell to the GM lineage conditioned on the fate of cell 5, at the center of the bidirectional chain (Fig. 6E-G). We found that the influence of the fate of cell 5 decreased with increasing distance from cell 5 in the chain. The probability that cell 2 commits to the GM lineage is only marginally affected by the fate of cell 5 (Fig. 6E). As a function of *A*_0_, over range of uncertainty in the fate of cell 5, cell 1 commits to the GM lineage with certainty. Cells near to cell 5, in contrast, are much more likely to commit to the GM lineage if cell 5 itself commits to the GM lineage (Fig. 6F,G). That is, local spatial architecture is captured in this model and manifests through the fate coupling of cells in close proximity. This is in stark contrast with cells signaling between each other in a fully connected topology, whereby cells all share the same distributions of cell-fate choice.

We expect hematopoietic organization in the bone marrow to reflect a niche intermediate between the two spatially extreme models studied here. As we have shown through simulation, spatial cell arrangements in the bone marrow will lead to a location-dependent coupling of cell fates in local neighborhoods of hematopoietic progenitor cells. This prediction of the reliance of cell fate on the fate of nearby cells is supported by experimental evidence. In recent work studying hematopoietic progenitor cells *in vivo*. Hérault et al. (2017) show that, unlike at steady state, during regeneration (when the requirement for new myeloid cell production is high), progenitor cells cluster together in groups. In agreement with our model, the spatial structure introduced by spatially proximal progenitor cells influences lineage commitment: the clustered GMPs activate cell cycle pathways and are driven towards differentiation into granulocytes, relative to the steady-state GMPs. Our results could be further tested in culture using similar methods to Hoppe et al. (2016) and analyzing the impact of spatial proximity on lineage commitment. Although it is currently beyond the capacity of experimental technologies to infer single-cell communication networks directly from data, this is changing (Giladi et al., 2020), leading to future opportunities to fit single-cell signaling models to data.

## DISCUSSION

Despite many theoretical and experimental advances in our understanding of gene regulatory network (GRN) dynamics, our ability to use GRN models to explain cell-fate decision-making during differentiation of multipotent progenitor cells remains limited. Here, with application to a well-studied cell fate GRN, the *GATA1-PU.1* mutual inhibition loop (Chickarmane et al., 2009; Chang et al., 2008; Roeder and Glauche, 2006), we introduced a new model that can simultaneously describe GRN dynamics and single cell-resolved cell-cell communication. Notably, although cell-cell communication is often assumed to be a crucial component of cell differentiation, it is rarely incorporated into models. The previous studies that have characterized cell-cell communication in models did not capture the detailed complexity of these dynamics, by making simplifying assumptions regarding either the GRN dynamics (Thurley et al., 2018) or the mechanisms by which cells signal (Smith and Grima, 2018; Mugler et al., 2016). We found that by combining these dynamic processes that describe noisy cell-fate decision-making at single-cell resolution, we were able to reconcile several outstanding controversies in the field.

Over a large domain of possible cell-cell communication topologies, we found that cell-cell communication alters cell-fate decision-making boundaries, which become probabilistic in response to the levels of external signaling factors. This helped to reconcile a controversy: that of whether or not transcriptional stochasticity is sufficient to initiate the granulocyte-monocyte versus megakaryocyte-erythrocyte cell-fate decision. Previous models supported the hypothesis; however, Hoppe et al. presented compelling evidence to contradict it (Hoppe et al., 2016). Our results agree with Hoppe et al., in that we show that eventual cell fate cannot be inferred from the initial gene expression state alone – and go further in emphasizing the stochastic nature of these cell-fate decisions (Moris et al., 2016). Analysis of cell-cell coupling led to the discovery that stable distributions of heterogeneous cell types can be robustly maintained by a set of external signals. This result offered insight into another open question: that of how population-level cell-fate behaviors emerge during cell differentiation (Mojtahedi et al., 2016). We also showed how (primarily extrinsic) noise increases the variability of cell-fate decision-making, in line with previous analyses of transcriptional noise during development (Mohammed et al., 2017). Finally, simulation of two topologies representing spatial extrema (well-mixed versus highly structured) suggest that increased coupling of cell fates occurs in proximal cells, a behavior observed experimentally during hematopoietic regeneration (Hérault et al., 2017).

We sought to tightly constrain model complexity here, for reasons of parsimony and interpretability. Relaxing some of the constraints imposed will likely yield further interesting results. We assumed throughout that cells were initially homogeneous, i.e. they shared the same initial conditions and internal GRN networks. Heterogeneous initial conditions and heterogeneous cell-fate decision-making (i.e. different GRNs in different cells) ought to be explored, e.g. by considering interactions between two progenitor cell types, each controlled by their own GRN. An alternative approach naturally lending itself to the analysis of spatially organized tissues and spatial heterogeneity is agent-based modeling. However, the methods that our framework relies on (tractability of the steady states and bifurcations) remain out of reach for most agent-based modeling approaches.

There is also much room for exploration of larger and more varied cell signaling network topologies. Here, future work should be guided by data, as it becomes harder to justify large signaling networks chosen *a priori*. Spatial transcriptomics (Moor and Itzkovitz, 2017) and new technologies (Giladi et al., 2020) will assist in the inference of cellular networks from data; inferred topologies could then be input to our model framework. These may include dissensus as well as consensus signals.

A central challenge for the model introduced here is that of fitting parameters to data. Ideally, this would require both spatially and temporally resolved single-cell transcriptomic data – at the limits of current technologies, although this is changing (Foreman and Wollman, 2020). Thus, in the current work we rely on comparison of qualitative features arising from the model with previous experimental studies. Moreover, due to the hybrid deterministic-stochastic formulation of the model, resulting in time-dependent signaling parameters, we doubt that it will be possible to derive a likelihood function for Bayesian parameter inference. Thus, approximate Bayesian computation will likely be required. Yet, even here, simulation times may be prohibitive or require further approximations to be made.

Future work should consider more thoroughly the stochastic nature of gene expression, e.g. by replacing the deterministic GRN dynamics with discrete stochastic simulation or stochastic differential equations (SDEs). This would be straightforward to accomplish numerically, although it will complicate the analysis of model bifurcations. Although the complete tractability of the deterministic model made it an ideal first candidate with which to study the effects of cell-cell communication, noise undoubtedly plays important roles in regulating single-cell phenotypes (Coomer et al., 2021).

In conclusion, the introduction of a single-cell resolved cell-cell communication model of GRN dynamics has helped to explain various cell-fate decision-making phenomena. Even in a tightly constrained model space, we have shown that changes in the distribution of cell fates due to cell-cell communication can be broad and varied. More generally, we have highlighted the need to consider multiscale effects in models of cell-fate dynamics. We anticipate that the application of these methods to other GRNs will lead to greater understanding of specific cell-fate decision-making control points, as well as general principles of control of stem cell differentiation.

## MATERIALS AND METHODS

### A multiscale model of cell-cell communication between single cells

To investigate how external signaling impacts intracellular dynamics during cell-fate decisions, we first must select an internal GRN topology to model. We choose to model a mutual inhibitory GRN: such network topologies arise frequently in cell-fate decision-making and developmental biology (Yu et al., 2018; Hong et al., 2015). Within certain parameter regions, such models give rise to two stable steady states and bistability (Fig. 1A). For example, it has been observed that the GRN determining the bifurcation of erythroid/megakaryocyte lineages from granulocyte/monocyte lineages contains a core mutual inhibitory loop topology consisting of mutual antagonism of *GATA1* (*G*) and *PU.1* (*P*). For this genetic switch, high expression of *G* corresponds to commitment to the erythroid/megakaryocyte lineage and high expression of *P* corresponds to commitment to the granulocyte/monocyte lineage.

This myeloid progenitor cell-lineage decision has been rigorously studied, and robust models exist of the intracellular GRN dynamics (Chickarmane et al., 2009; Duff et al., 2012; Chang et al., 2008; Huang et al., 2007; Roeder and Glauche, 2006; Strasser et al., 2012). Here, we implement the ODE model defined by Chickarmane et al., which follows the Shea-Ackers formalism for transcription factor dynamics (Shea and Ackers, 1985; Chickarmane et al., 2009). The ODE model is given by Eqns 1-3, where *X* is a transcription factor recruited by GATA1 to bind to and inhibit PU.1 expression. These ODEs give rise to two stable steady states for a region of parameter space with respect to the parameters *A*, *B* and *C*, where *A*, *B* and *C* are parameters summarizing environmental signals (Fig. 1B). The parameters through which the extracellular environment is summarized will be modified below to implement a multiscale model of cell-cell communication. The parameter values used throughout this work can be found in Table S1.
(1)

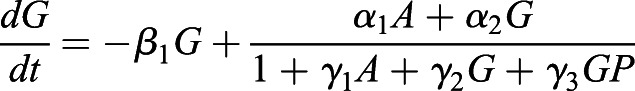

(2)

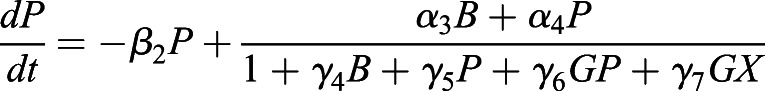

(3)

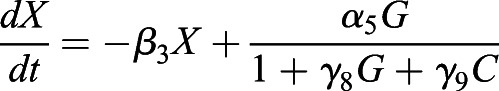
To incorporate cell-cell signaling into the model, we must modify the cell-internal ODEs in a way that reflects the signal received by the cell without unrealistically changing the internal dynamics (e.g. a loss of bistability). For simplicity, we choose a single parameter *A* to summarize the effects of external signaling. We study both signals that recruit nearby cells to commit to the same lineage and the opposite lineage of the sender. For the first case, we consider two cells, cell 1 and cell 2, where cell 1 signals to cell 2. Let *G*_1_(*t*) and *P*_1_(*t*) denote the concentrations of *G* and *P* in cell 1 at time *t*, respectively, and let *A*_2_(*t*) be the value of the parameter *A* in cell 2 at time *t*. As cell 1 signals to cell 2 to coordinate fate decisions (‘be like me’ signal), then *A*_2_(*t*) should increase if the ratio *G*_1_(*t*):*P*_1_(*t*) indicates that cell 1 has committed to – or is increasingly likely to commit to – the ME state (where *G* is highly expressed). Conversely, *A*_2_(*t*) should decrease if the ratio *G*_1_(*t*):*P*_1_(*t*) indicates that cell 1 has committed to – or is increasingly likely to commit to – the GM state where *P* is highly expressed. To model the arrival process of the signals received by cells, we use a Poisson process. This choice is based upon previous results, which have demonstrated that the arrival of independent Brownian particles to an absorbing boundary is a Poisson process (Nadler et al., 2001). This result does assume that ligands are continuously produced and secreted from the ‘sender’ cell, which does not fully reflect the underlying biology, but we reason that it is an appropriate limiting assumption to allow for a simple, tractable signaling model. Based on these assumptions, we constructed a communication model: we treat signaling as a Poisson process, where the wait time between signals being sent by a cell follows an exponential random variable with mean *μ*. After this wait time, at time *t*_*s*_, a signal is sent and we change the value of *A*_2_ by:
(4)

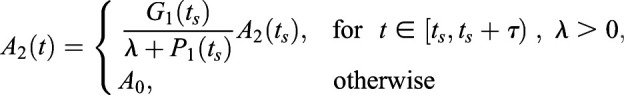
After a delay, *τ*, *A*_2_ returns to its original value, *A*_0_ (Fig. 1C). Returning to the same value *A*_0_ between signals ensures that attractor states (Fig. 1B) do not change. Sampled wait time values are rounded to integer values to avoid integration errors. In Fig. 1D is a schematic of this signaling model. Fig. S9 gives a sample simulation of a chain of four cells and the values of *A*_2_, *A*_3_ and *A*_4_ plotted over time. Eqn 4 defines a signal between cells 1 and 2, but holds equally in the case of any signal between cells *l* and *k*.

In the case of signals that promote nearby cells to commit to the opposite lineage, we can follow the same principles to define a signal:
(5)

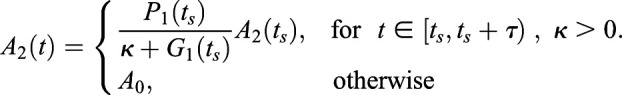
These definitions of signaling also allow for multiple cells to signal a single cell at similar times, while still having *A*_2_ well defined. We implemented this model in Julia (Bezanson et al., 2017), where we used the DifferentialEquations.jl (Rackauckas and Nie, 2017) and Distributions.jl (Besançon et al., 2021) packages for numerical simulation.

The model developed here shares similarities with models specified by piecewise-deterministic Markov processes (PDMPs), first introduced by Davis (Davis, 1984). Such models comprise continuous dynamics and a discrete Markov process that can alter the continuous dynamics at discrete time points. PDMPs have previously been applied to biological systems for the study of gene expression and genetic networks (Rudnicki and Tyran-Kamińska, 2017; Zeiser et al., 2008). In the case of two cells sharing one directed signal between them, the model described here closely resembles a PDMP, with the exception that here, due to information transfer between cells, the discrete process is not Markovian.

### Model specification and choice of parameters

To elucidate our choice of signaling model, we briefly discuss some alternatives. Examples of alternative signaling model choices include: signals that change more than one ODE parameter, additive rather than multiplicative signaling, or signals that permanently change ODE parameters. We choose to modify a single parameter for interpretability and to constrain the model space: the ODE system exhibits bifurcations with respect to many of its parameters, and we do not seek to explore bifurcations of co-dimension 2 or greater here. Modeling with additive signals brings complications, such as the possibility of obtaining negative values of *A*. In addition, if a large number of committed cells are communicating with a single cell, the value of |*A*| may be unrealistically large and dominate the ODE. This problem is dealt with in the signaling model we present here through the parameter *λ*. Last, permitting signals to cause permanent changes to ODE parameters (rather than over time intervals) can result in the divergence of *A*. Thus, we have selected a signaling model that can capture how changes in the extracellular environment can alter cell internal GRN dynamics while still preserving the overall behaviors (i.e. the attractor states) of the dynamical system.

In this work, we will assume that cells are homogeneous, i.e. all cells share the initial conditions (*G*, *P*, *X*)=(20, 1, 20). The internal GRNs are identical with the same parameter values, including the same initial value of *A*, denoted *A*_0_. We set the signaling period to be *τ*=5, and the mean of the signaling wait time distribution to be *μ*=50. Changing the values of *μ* and *τ* does cause qualitative changes as long as *τ* is sufficiently large relative to *μ*. For more insight on the relationship between the wait time distribution and *τ*, see Fig. S10. Unless otherwise specified, we set *λ*=*κ*=1. We initially chose *λ*=*κ* =1 to avoid unrealistic behaviors when *G* or *P*≈0. We will provide an in depth discussion on the roles of these parameters in the next section. For all topologies depicted schematically in Fig. 3, regular arrowhead arrows correspond to consensus signaling (Eqn 4) and flat (inhibition) arrowhead arrows correspond to dissensus signaling (Eqn 5).

As a result of signaling, cell-fate decisions became probabilistic rather than deterministic. Then, for each cell in a signaling topology, we can examine the probability distribution of the cell converging to a certain lineage. To do so, for each signaling topology tested, we selected a range of values of *A*_0_ to simulate. The step size between the *A*_0_ values was scaled between each topology, depending on the range of the probability distribution. For each value of *A*_0_, we simulated *N* trajectories, counting the number of times each cell converged to both fates. From these counts, we estimated the probability that each cell converged to the *G* high state and plotted:

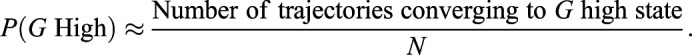
A sample of the simulated data points can be found in Fig. S11. Unless stated otherwise, probabilities are estimated by running *N*=1000 simulations.

While *λ*=1 was an intuitive first choice to avoid divergence in signaling parameters, it relies on the assumption that a cell converging to the *P* high state implies that *P* is more highly expressed than *G*+1, and vice versa. This turns out to not necessarily be true. Rather, for a given value of *A*_0_, being in the *P* high state means that *P* is more highly expressed relative to the other stable steady state value of *P*. Recalling Fig. 1B, we see that for some values of *A*_0_, e.g. *A*_0_=0.8, *G* is always more highly expressed than *P* regardless of which stable steady state a cell converges to. Looking at the bifurcation diagram, we see that in the *P* high state, the maximum steady state value of *G* is G*≈16.97 and the minimum value of *P* is P*≈1.475. Similarly, in the *G* high state, the maximum steady state value of *P* is P*≈0.3529 and the minimum value of *G* is G*≈40.35. From here, we wanted to select values of *λ* that satisfy the following:

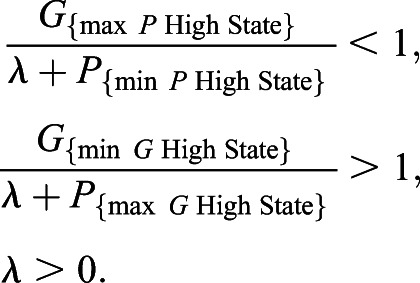
These inequalities are satisfied for *λ*∈[15.495, 39.997]. Similarly, for *κ*, in order to satisfy the analogous inequalities for the dissensus signal, we redefine the dissensus signal as
(6)

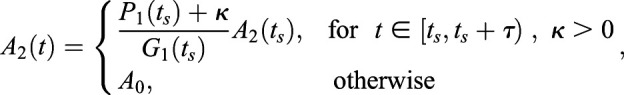
where *κ*∈[15.495, 39.997]. We will explore how selecting values of *λ* in or near this range changes the behaviors of both individual cells and populations of cells.

### Intrinsic and extrinsic cell-cell communication noise

In this work, we focus on how intercellular signaling alters cell decision making. Therefore, we only consider noise with respect to signaling (not the internal GRN dynamics). Noise in the signal can arise due to the noisy extracellular environment (extrinsic noise) or can arise during the signal transduction within a cell (intrinsic noise) (Smith and Grima, 2018; Thurley et al., 2018). Here, we define models for both sources of noise.

For extrinsic noise, i.e. noise with respect to the extracellular environment, let *η*_*e*_ be a random variable such that 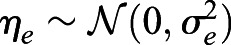, where 
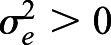
. For cell *k* in a given topology, at the start of each new wait period, we sample a value of *η*_*e*_ and update *A*_*k*_(*t*) by
(7)

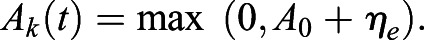
We truncate *A*_*k*_(*t*) at zero to avoid negative values. However, we select values of 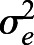 small enough that the truncation is rarely necessary in simulation. Note that noise in *A*_*k*_(*t*) also results in noise during the signaling period according to Eqns 4 and 5.

For intrinsic noise, i.e. noise with respect to signal transduction, let *η*_*p*_ be a random variable such that 
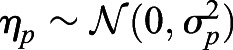
, where 
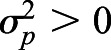
. For cell *k* in a given topology, at the start of each new pulse period, we sample a new value of *η*_*p*_ and update *A*_*k*_(*t*) as follows:
(8)

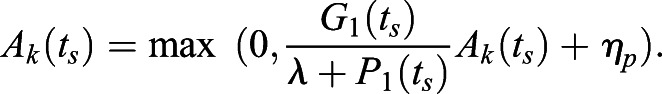
In the analysis of noise effects, we decrease the mean wait time to *μ*=40, resulting in a larger signal-to-wait time ratio, allowing signals to have a greater influence on target cells. Doing so accentuates the effects of intrinsic and extrinsic noise because cells are spending more time in a pulse state.

## Supplementary Material

Supplementary information

Reviewer comments
