# Supplementary material for: A single-cell resolved cell-cell communication model explains lineage commitment in hematopoiesis
Source: Development. 2021 Dec 22;148(24):dev199779. doi: 10.1242/dev.199779 (PMC8722395; doi:10.1242/dev.199779)
Supplement: Supplementary information [file develop-148-199779-s1.pdf]

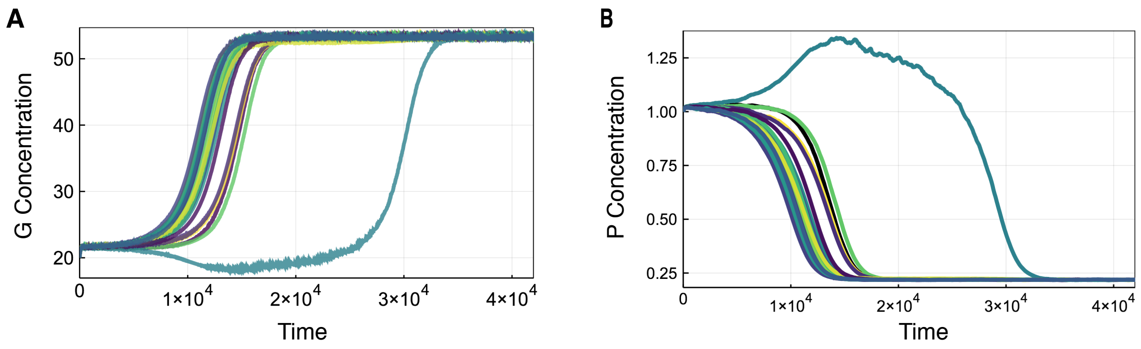

Fig. S1. Sample simulation of a loop of 20 cells with concentrations of both G *and* P over time. Parameters are set to $\lambda = 28.0$ and $A_0 = 1.0$.

Development • Supplementary information

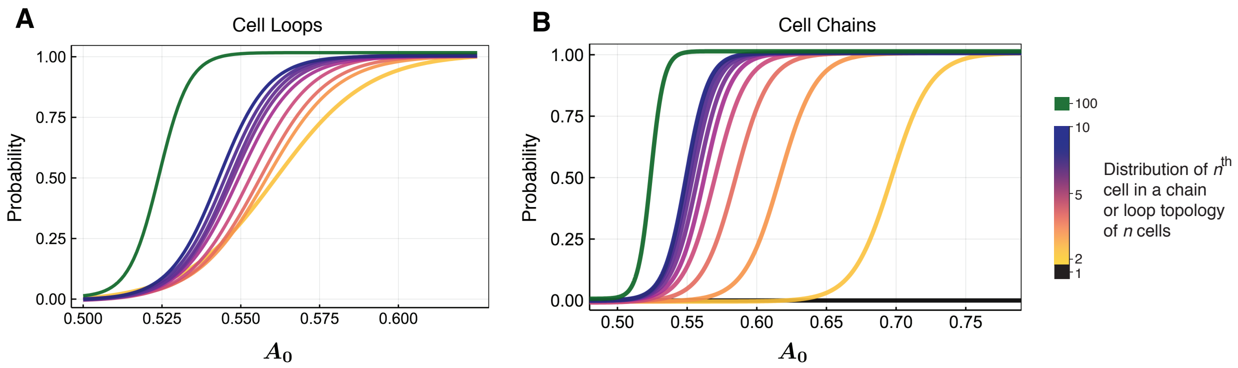

Fig. S2. Chains and loops of cells from Fig. 3A-3B along with curves where the number of cells $n = 100$. The number of simulation iterations was reduced to 100 per A_0 value in simulations of 100 cell topologies.

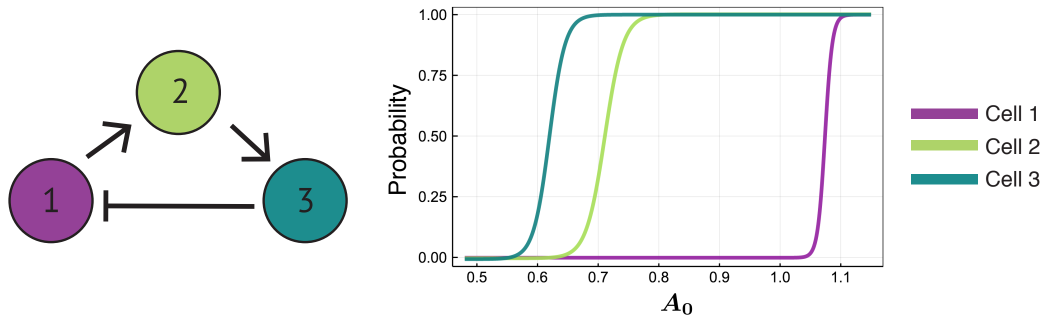

Fig. S3. Cell fate probability distributions for a loop of three cells with one inverse signal.

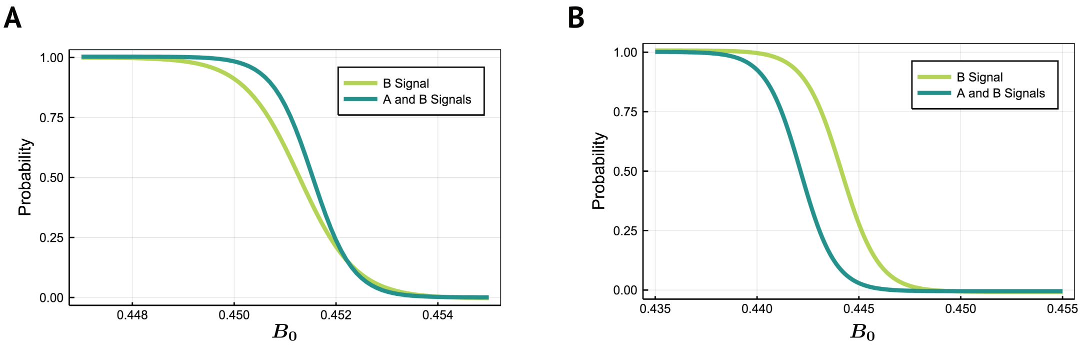

Fig. S4. Probability distributions of (A) cell two in a chain and (B) two cell loop converging to the G high state resulting from a consensus signal to the parameter B and dual consensus signals to both parameters A and B. A_0 is fixed as 0.65. The signaling parameters for the signals changing A and B are $\lambda_A = 28.0$ and $\lambda_B = 28.0$ respectively.

Development • Supplementary information

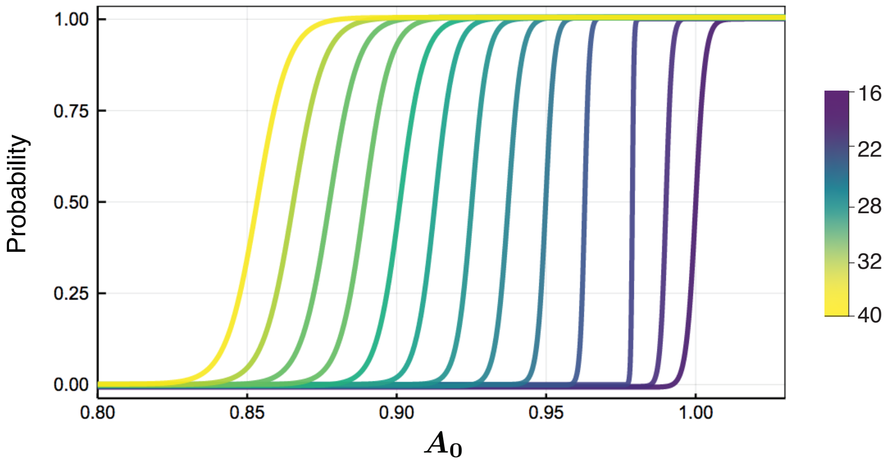

Fig. S5. Probability distributions of cell fate commitment to G high steady state for cell 2 in a chain of two cells with a dissensus signal where $\kappa \in [16, 40]$.

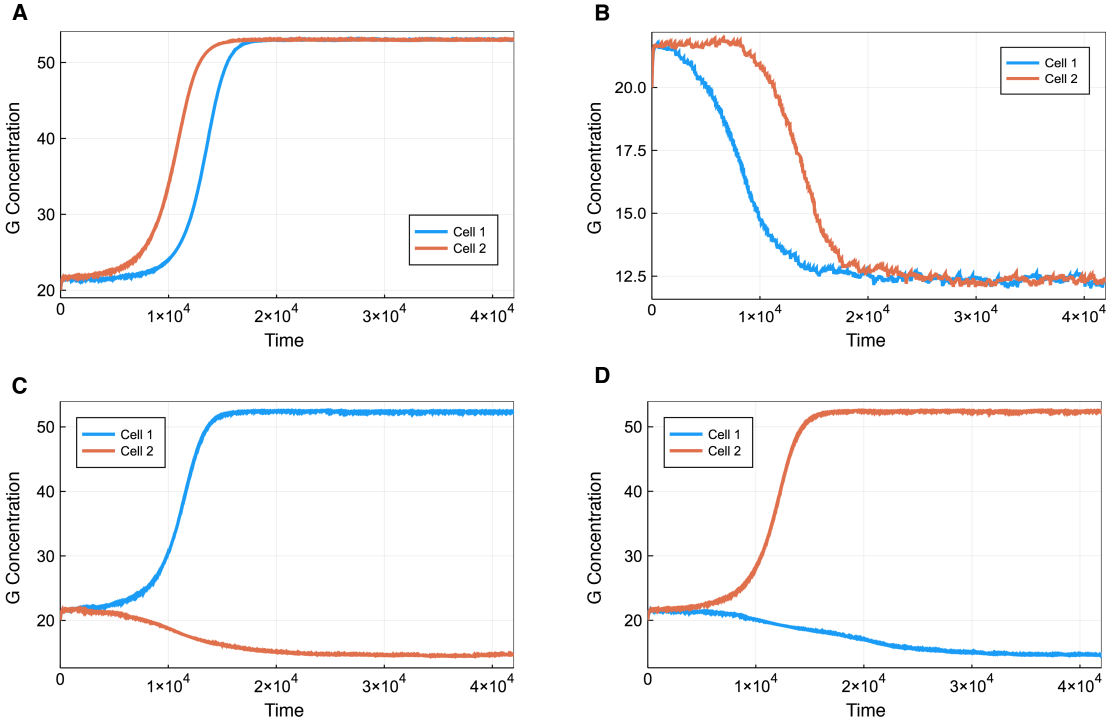

Fig. S6. (A)-(D) Sample trajectories of a loop of two cells with $A_0 = 1.0175$ and $\lambda = 40$.

Development • Supplementary information

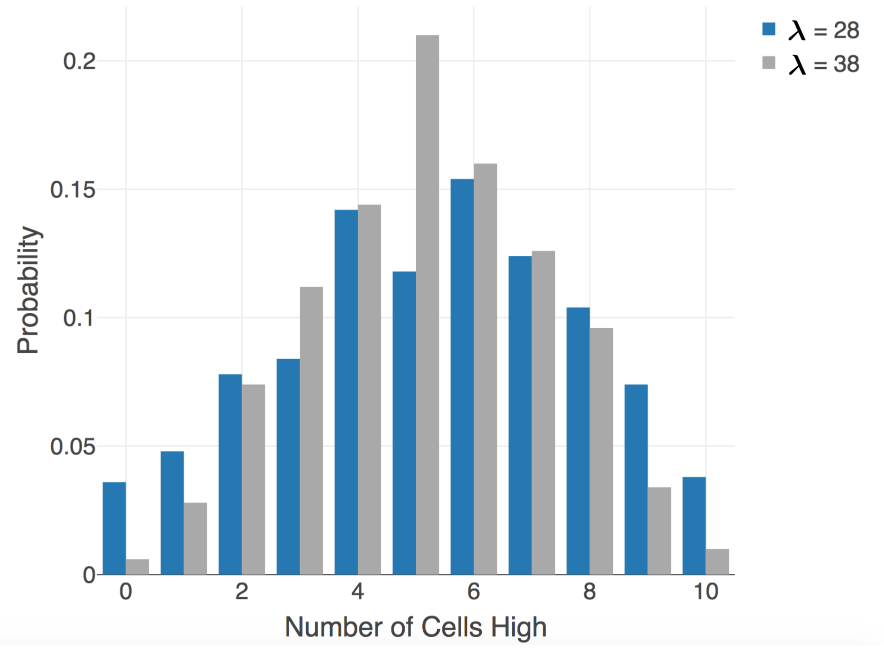

Fig. S7. Distributions of loops of 10 cells with different values of λ. The values of A_0 were selected so that the distributions had similar expected values. For the blue distribution, $A_0 = 0.9973$ and the expected value is 5.284. For the grey distribution, $A_0 = 1.016$ and the expected value is 5.154

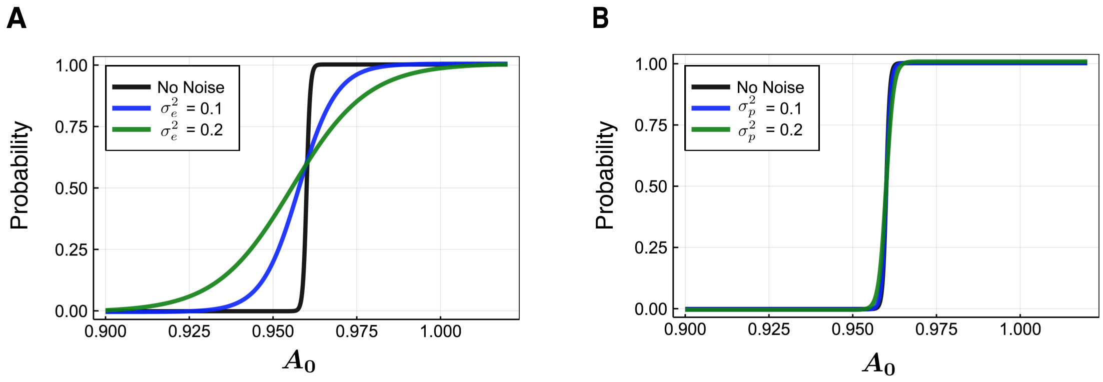

Fig. S8. Probability distributions that a given cell in a two cell loop will converge to the erythroid state with varying amounts of (A) extrinsic noise or (B) intrinsic noise.

Development • Supplementary information

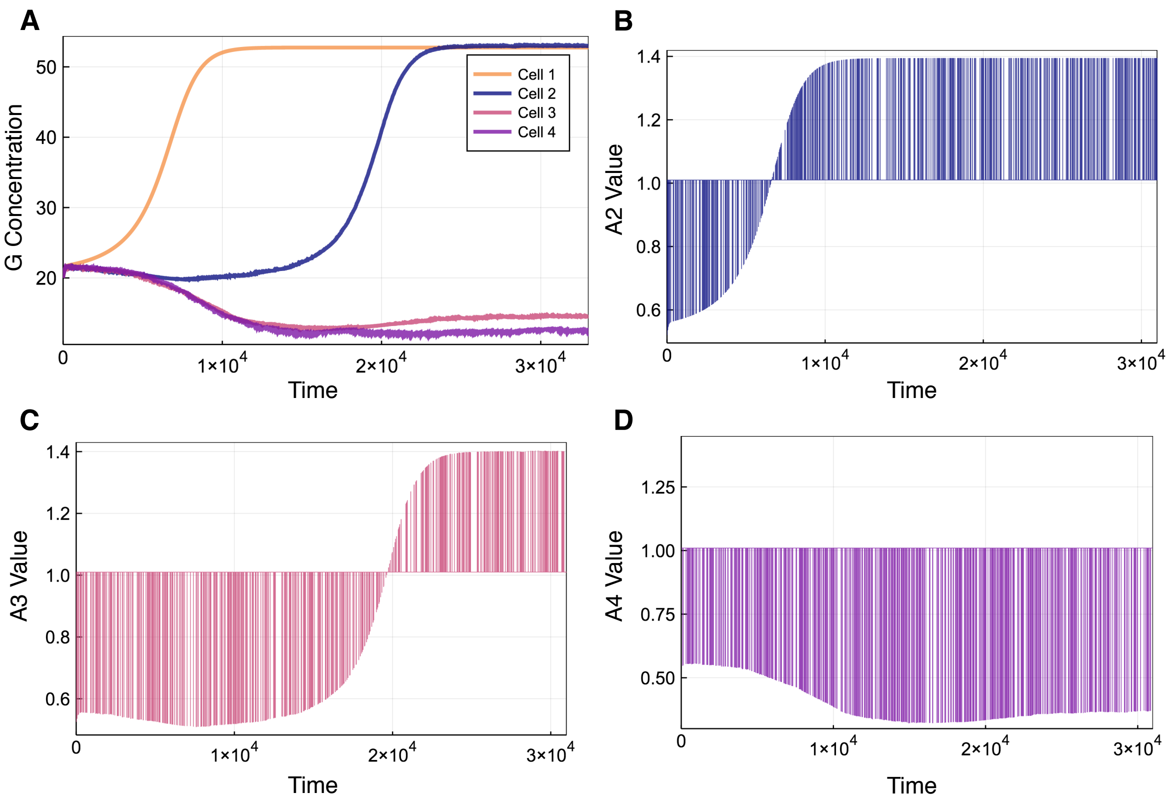

Fig. S9. (A) Sample trajectory of a chain of four cells where $A_0 = 1.01$ and $\lambda = 38.0$. (B)-(D) Plots of $A_2(t)$, $A_3(t)$, and $A_4(t)$ over the same timescale as the trajectory.

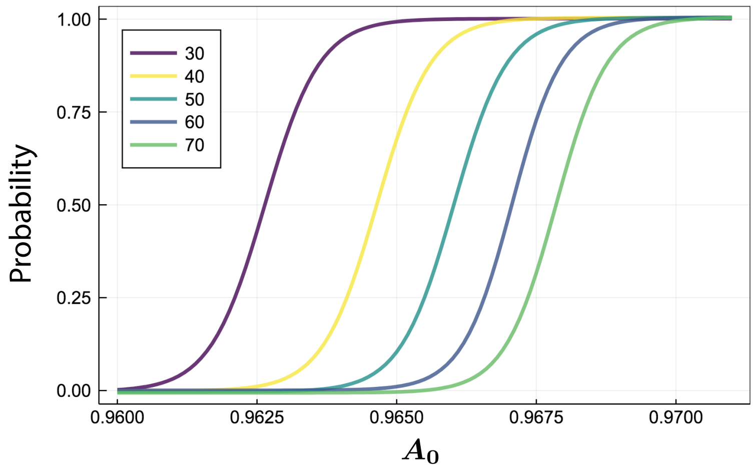

Fig. S10. Probability distributions of cell 2 in a chain of cells converging to the G high state where $\lambda = 18$ with different mean wait times, μ, given in the legend.

Development • Supplementary information

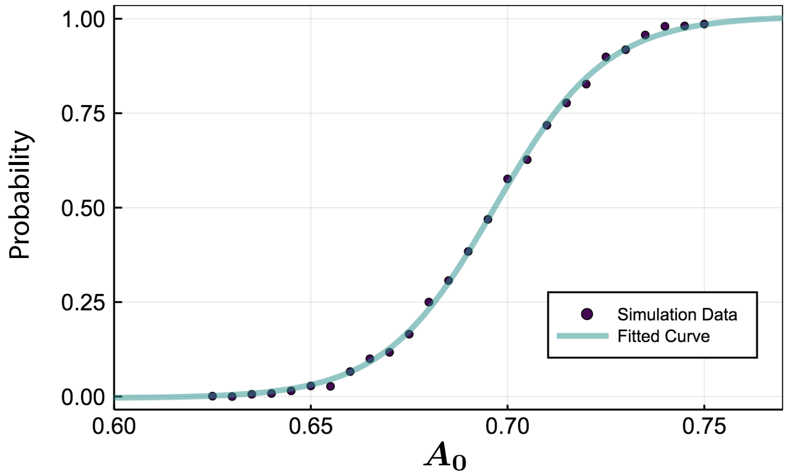

Fig. S11. Sample of simulated data points along with the fitted curve for cell 2 in a chain of cells where $\lambda = 1$.

Table S1. Parameter values used in the ODE system.

α_1	α_2	α_3	α_4	α_5	β_1	β_2	β_3	γ_1	γ_2
1.0	0.25	1.0	0.25	0.01	0.01	0.01	0.01	1.0	0.25
γ_3	γ_4	γ_5	γ_6	γ_7	γ_8	γ_9	B	C	
1.0	1.0	0.25	1.0	0.13	0.01	10.0	0.5	0	
