# Supplementary material for: A single-cell resolved cell-cell communication model explains lineage commitment in hematopoiesis
Source: Development. 2021 Dec 22;148(24):dev199779. doi: 10.1242/dev.199779 (PMC8722395; doi:10.1242/dev.199779)
Supplement: Reviewer comments [file develop-148-199779_review_history.pdf]

A single-cell resolved cell-cell communication model explains lineage commitment in hematopoiesis

Megan K Rommelfanger and Adam L MacLean
DOI: 10.1242/dev.199779

Editor: Paul Francois

Review timeline

Original submission:	3 May 2021
Editorial decision:	9 July 2021
First revision received:	11 October 2021
Accepted:	6 November 2021

Original submission

First decision letter

MS ID#: DEVELOP/2021/199779

MS TITLE: A single-cell resolved cell-cell communication model explains lineage commitment in hematopoiesis

AUTHORS: Megan K Rommelfanger and Adam L MacLean

I hope you and your family are well.

I have now received all the referees' reports on the above manuscript, and have reached a decision. The referees' comments are appended below, or you can access them online: please go to BenchPress and click on the 'Manuscripts with Decisions' queue in the Author Area.

As you will see, the referees express considerable interest in your work, but have some significant criticisms and recommend a substantial revision of your manuscript before we can consider publication. If you are able to revise the manuscript along the lines suggested, which may involve further experiments, I will be happy receive a revised version of the manuscript. Your revised paper will be re-reviewed by one or more of the original referees, and acceptance of your manuscript will depend on your addressing satisfactorily the reviewers' major concerns. Please also note that Development will normally permit only one round of major revision.

We are aware that you may be experiencing disruption to the normal running of your lab that make experimental revisions challenging. If it would be helpful, we encourage you to contact us to discuss your revision in greater detail. Please send us a point-by-point response indicating where you are able to address concerns raised (either experimentally or by changes to the text) and where you will not be able to do so within the normal timeframe of a revision. We will then provide further guidance. Please also note that we are happy to extend revision timeframes as necessary.

Please attend to all of the reviewers' comments and ensure that you clearly highlight all changes made in the revised manuscript. Please avoid using 'Tracked changes' in Word files as these are lost in PDF conversion. I should be grateful if you would also provide a point-by-point response detailing how you have dealt with the points raised by the reviewers in the 'Response to Reviewers' box. If you do not agree with any of their criticisms or suggestions please explain clearly why this is so.

© 2021. Published by The Company of Biologists under the terms of the Creative Commons Attribution License (https://creativecommons.org/licenses/by/4.0/).

Reviewer 1

Advance summary and potential significance to field

The paper propose a toy model for cell-cell signalling that on top of a bistable switch network is capable of inducing different heterogeneous population of differentiating cell types in different geometries. I found interesting the use of a different family of models -- Poisson communication -- that could provide a novel dynamical frameworks to understand cell signalling.

Comments for the author

My main concern with the manuscript is that in spite of the mathematical appeal of using a Poisson model for cell-cell communication, the study was too detached from biological mechanisms or questions:
1) There is no explanation for the choice of the Poisson model. What is the biological mechanism by which cells enter and exit some sort of receiver state? In which limit such a process emulates a diffusing signal? For many simulations the interaction between cells was asymmetric, what was the rationale behind that choice?
2) Most of the simulations of the paper, if I understood correctly, take place in cells interacting in very specific spatial configurations: loops and cascades.
This is a completely different scenario to myeloid progenitor cells either in vitro or in vivo. I would also expect that the interactions described in the model produce very particular spatial patterns, are these patterns observed in vivo?
3) The authors claim that this reconciles traditional models with the observations that initial levels of GATA1 and PU.1 do not determine cell fate. But such heterogeneity could come from much more simpler mechanisms such as extrinsic noise in the signals e.g. noise in one of the parameters or other unidentified transcription factors. This contrasts with the model proposed in the paper: there is no clear evidence on the necessity of such complex cell-cell communication mechanism for a system in which such cell-cell communication has not been experimentally observed. Is there any observation of the model in relation to experimental evidence that could not be explained without cell-cell communication or without that particular description of stochasticity?

Reviewer 2

Advance summary and potential significance to field

The authors develop a cell fate model of hematopoiesis that couples external communication between cells in addition to the internal gene regulatory network of each cell. The gene regulatory network model includes GATA1 and PU.1 that describes the hematopoiesis differentiation to megakaryocyte-erythroid (ME) lineage or granulocyte-monocyte (GM) lineage. Also, the model includes parameters that summarize the cell-cell communication signals or environmental signals. The authors focus on studying the stochasticity in the signals and demonstrate that the noise yields non-deterministic cell fate decisions. In particular, the model is studied with respect to varying base signal strength A_0, cell-cell coupling parameter λ, and intrinsic and extrinsic noise in signaling. The authors carefully study multiple cell communication topologies and demonstrate that the initial gene expression alone cannot determine the fate that agrees with results in Hoppe et al. Also, the extrinsic noise is shown to contribute more than the intrinsic noise that agrees with Filippi et al.

The manuscript nicely demonstrates the importance of stochastic modeling that supports recent biological findings.

Comments for the author

1. I think two arrows in Figure 1D does not match the equations, the arrow between G and X (arrowhead?), and arrow between X and P (inhibition?).
2. In figure 3, I observe that the probability curve becomes more like a step function as n increases. What will happen if we increase n even further like 100 or

© 2021. Published by The Company of Biologists under the terms of the Creative Commons Attribution License (https://creativecommons.org/licenses/by/4.0/).

more?
3. The authors carefully considered various topologies as in Figure 3. However, I wonder which of them are realistic and how can one extend those to the actual hematopoiesis system. I regard hematopoiesis as a well mixing system rather than having a fixed topology between cells. Also, I believe the spatial component would be important. Do you think if we consider something like a three dimensional agent based model, similar conclusion would hold?
4. Can you provide some biological examples of consensus signaling and inhibition signaling of ME and GM? I think it would help biologist to further design experiments and measure those signals.
5. I understand that for simplicity, only A is modified to incorporate external signaling, but I wonder what effects will considering noise in all B and C have. Will it add more stochasticity or cancel each other to stabilize the noise effect?
6. In the subsection on page 8, when you discuss the importance of parameter λ, will altering κ have similar effects? Can you comment on that?
7. I wonder if the results without the extrinsic noise in the signaling is significant enough. In Figure 3, I see the probability becoming closer to a step function as the number of cell n increases which would be closer to reality, and in Figure 4, the interesting A_0 seems to be a very tuned value that happens by changing A_0 from 1 to 1.002, which is 0.2% of the base value.
8. Minor comments:

- In the end of page 6, after 'decrease wait time to $\mu = 40$', can you elaborate on 'order to distinguish between the effects'?
- In Figure 5B, if you plot the lines with the same x axis scale as in C and D, I think it would be more straightforward to compare, but I will the decision to you.
- In page 7, testing 20 cell loop topology, can you refer to Figure 3 where you have the figure of cell topology?
- In page 4, why don't you merge the discussion of introducing λ, κ values as one ($\lambda = \kappa = 1$) and explaining how to choose the range as you did in page 5.

First revision

Author response to reviewers' comments

Response to Reviewer 1

The paper propose a toy model for cell-cell signalling that on top of a bistable switch network is capable of inducing different heterogeneous population of differentiating cell types in different geometries. I found interesting the use of a different family of models -- Poisson communication -- that could provide a novel dynamical frameworks to understand cell signalling.
My main concern with the manuscript is that in spite of the mathematical appeal of using a Poisson model for cell-cell communication, the study was too detached from biological mechanisms or questions:

Comment 1: There is no explanation for the choice of the Poisson model. What is the biological mechanism by which cells enter and exit some sort of receiver state? In which limit such a process emulates a diffusing signal? For many simulations the interaction between cells was asymmetric, what was the rationale behind that choice?

Response: We thank the reviewer for their positive comments on the appeal of our work, and appreciate that in places we had not previously described in sufficient detail the biological rationale behind model assumptions. In the revision we have carefully gone over each of the assumptions and added details where needed. In particular, regarding the Poisson signaling model, previous work has shown that the arrival of independent Brownian particles to an absorbing

© 2021. Published by The Company of Biologists under the terms of the Creative Commons Attribution License (https://creativecommons.org/licenses/by/4.0/).

boundary is Poissonian [1].

The result in [1] is relevant in this case since we assume that cells homogeneously express surface receptors above a certain threshold, i.e. the cell is always in a receiver state, and that diffusible ligands can bind to these receptors at discrete times, i.e. arriving independently.
Ligands diffuse through extracellular space – thus we think the binding assumption is well justified. The result of [1] however does also assume that ligands would be constantly produced and secreted from the "sender" cell. This of course does not accurately reflect the underlying biology, but we reason that it is an appropriate limiting assumption to allow for a simple, tractable signaling model.

We have added the discussion and justification of the choice of the Poisson distribution in the cell-cell communication model to the main text in the Methods section.

> "To model the arrival process of the signals received by cells, we use a Poisson process. This choice is based upon previous results which have demonstrated that the arrival of independent Brownian particles to an absorbing boundary is a Poisson process (Nadler et al., 2001). This result does assume that ligands are continuously produced and secreted from the "sender" cell, which does not fully reflect the underlying biology, but we reason that it is an appropriate limiting assumption to allow for a simple, tractable signaling model."

Comment 2: Most of the simulations of the paper, if I understood correctly, take place in cells interacting in very specific spatial configurations: loops and cascades.
This is a completely different scenario to myeloid progenitor cells either in vitro or in vivo. I would also expect that the interactions described in the model produce very particular spatial patterns, are these patterns observed in vivo?

Response: We appreciate this important point, also discussed by reviewer 2, regarding investigation into the relationships between spatial patterns and cell fate determination. The loops and cascades were chosen as they represented the most basic repeatable patterns of cell signaling, however there is not an obvious 1:1 mapping between these topologies and spatial architecture.

To connect signaling topologies directly to spatial architecture, in the revision we have performed new analyses of topologies that explicitly represent space through their signaling configurations. We analyzed two new signaling topologies. The first of these is a fully connected topology: it represents cells that do not occupy spatially restricted niches, i.e. they can be considered to be in a well-mixed environment. The alternative topology is a bidirectional cell chain: representing a spatially structured model where cells can only interact proximally with their nearest neighbors. The differences in cell fate commitment under these topologies was striking: whereas well-mixed cells are equally dependent on the fate choices of all other cells (as we would expect), in the case of the bidirectional cell chain, cell proximity strongly influenced fate commitment. These topologies represent two possible extremes: no spatial structure or highly spatially structured. Hematopoietic progenitor cells *in vivo* are likely in an intermediate arrangement. By characterizing the limits of spatially-extreme models we are able to understand the possible range of cell fate behaviors that they can produce.

We discuss these results in detail in the revised manuscript in a new Results section ("Spatial structure in communication networks impacts cell fate outcomes") and a corresponding new figure (Fig. 6).

These results predict a reliance of cell fate on the fate of nearby cells: this is supported by experimental evidence. In recent work detailing spatial locations of hematopoietic progenitor cells *in vivo*, Hérault et al. [2] show that, unlike at steady state, during regeneration (when requirement for new myeloid cell production is high) progenitor cells cluster together in groups. In agreement with our model, the spatial structure introduced by spatially proximal progenitor cells influences lineage commitment. Hérault et al. show that clustered GMPs activate cell cycle pathways and are driven towards differentiation into granulocytes, relative to the steady state GMPs. While it is currently beyond the scope of experimental technologies to directly infer single-cell communication networks from *in vivo* data, this is changing [3], and we are excited for future opportunities to infer signaling models directly from data.

© 2021. Published by The Company of Biologists under the terms of the Creative Commons Attribution License (https://creativecommons.org/licenses/by/4.0/).

Comment 3: The authors claim that this reconciles traditional models with the observations that initial levels of GATA1 and PU.1 do not determine cell fate. But such heterogeneity could come from much more simpler mechanisms such as extrinsic noise in the signals e.g. noise in one of the parameters or other unidentified transcription factors. This contrasts with the model proposed in the paper: there is no clear evidence on the necessity of such complex cell-cell communication mechanism for a system in which such cell-cell communication has not been experimentally observed. Is there any observation of the model in relation to experimental evidence that could not be explained without cell-cell communication or without that particular description of stochasticity?

Response: We appreciate the reviewer's request to further justify the mechanism of cell-cell signaling as the driver of cell fate specification. This is an important point, and while experimental technologies cannot yet directly measure cell-cell interactions linked to fate outcomes in vivo, multiple lines of evidence suggest that extrinsic noise alone is not sufficient to dictate hematopoietic cell fates, i.e. that cell-cell communication is necessary. We discuss two key sources.

1. In vivo, it has been shown that the number of hematopoietic cells transplanted affects the phenotype of the regenerated blood system in the recipient [4]. Greater numbers of cells lead to more balanced production of myeloid and lymphoid lineages; when fewer cells are transplanted the lineages are biased towards specific subsets of lymphocytes. Since the extracellular environment (the bone marrow of the recipient) is the same in these experiments, and the minimum cell numbers (100-400) are well above the limit in which we would expect extrinsic noise to dominate, cell-cell communication between hematopoietic cells is the most likely mechanism to explain the differences between the phenotypes observed.

2. In vitro, stem cell—niche interactions can be more tightly controlled and quantified. In a carefully designed microcavity system, hematopoietic stem and progenitor cells were placed into wells of different cavity sizes (15 or 40 μm) [5]. This study found that cell fate (long-term vs short-term HSC behavior) was controlled by the size of the cavity. Given that the size of murine hematopoietic stem/progenitor cells is approximately 5-10 μm, the smaller wells likely contain only 1-2 cells and individual cells will experience many fewer (if any) external signals from neighbors, compared with cells in the larger wells. Given that all other external factors are held constant across these experiments, the additional signaling that cells can receive in the case of the larger wells is the most probable source of the differences in hematopoietic cell fate.

We discuss this experimental evidence for the influence of cell-cell communication on cell fate in the Introduction of the revised manuscript.

We also note -- with regard to the question of whether or not similar results could be obtained via simpler mechanisms of extrinsic noise -- that this can be assessed in light of the new results on spatial modeling (See answer to Comment 2 above). Here, we explicitly looked at cell fate conditional probabilities, i.e. the dependence of a cell's fate on its spatial location in a signaling network. We showed that the conditional probabilities vary between cells. Such results could not be obtained in a model that only considered extrinsic noise, i.e. that excluded cell-cell interactions.

Response to Reviewer 2

The authors develop a cell fate model of hematopoiesis that couples external communication between cells in addition to the internal gene regulatory network of each cell. The gene regulatory network model includes GATA1 and PU.1 that describes the hematopoiesis differentiation to megakaryocyte-erythroid (ME) lineage or granulocyte-monocyte (GM) lineage. Also, the model includes parameters that summarize the cell-cell communication signals or environmental signals. The authors focus on studying the stochasticity in the signals and demonstrate that the noise yields non-deterministic cell fate decisions. In particular, the model is studied with respect to varying

© 2021. Published by The Company of Biologists under the terms of the Creative Commons Attribution License (https://creativecommons.org/licenses/by/4.0/).

base signal strength A_0, cell-cell coupling parameter λ, and intrinsic and extrinsic noise in signaling. The authors carefully study multiple cell communication topologies and demonstrate that the initial gene expression alone cannot determine the fate that agrees with results in Hoppe et al. Also, the extrinsic noise is shown to contribute more than the intrinsic noise that agrees with Filippi et al. The manuscript nicely demonstrates the importance of stochastic modeling that supports recent biological findings.

In this paper, the authors develop a cell fate model of hematopoiesis that couples external communication between cells in addition to the internal gene regulatory network of each cell. The gene regulatory network model includes GATA1 and PU.1 that describes the hematopoiesis differentiation to megakaryocyte-erythroid (ME) lineage or granulocyte-monocyte (GM) lineage. Also, the model includes parameters that summarize the cell-cell communication signals or environmental signals. The authors focus on studying the stochasticity in the signals and demonstrate that the noise yields non-deterministic cell fate decisions. In particular, the model is studied with respect to varying base signal strength A0, cell-cell coupling parameter λ, and intrinsic and extrinsic noise in signaling. The authors carefully study multiple cell communication topologies and demonstrate that the initial gene expression alone cannot determine the fate that agrees with results in Hoppe et al. Also, the extrinsic noise is shown to contribute more than the intrinsic noise that agrees with Filippi et al. The manuscript nicely demonstrates the importance of stochastic modeling that supports recent biological findings. I would recommend accepting the paper after the authors discuss the following comments.

Response: We thank the reviewer for their enthusiastic comments regarding this work. We appreciate the constructive comments, which are addressed point-by-point below.

Comment 1: I think two arrows in Figure 1D does not match the equations, the arrow between G and X (arrowhead?), and arrow between X and P (inhibition?).

Response: We appreciate the reviewer catching this. Indeed, the equations were correct but the figure was erroneous: X is activated by G, and X inhibits P. This has been corrected.

Comment 2: In figure 3, I observe that the probability curve becomes more like a step function as n increases. What will happen if we increase n even further like 100 or more?

Response: Indeed, as the number of signals received by a cell increases, so does the fidelity of its response (the steepness of the curve). In addition: large loops or chains of cells approach the same limiting distribution (sharp sigmoidal curve) for an n-cell loop or the nth cell in a chain. To analyze the fate probability distributions of large signaling topologies rigorously, we performed new simulations of larger topologies: 100 cells interacting either in a chain or a loop. Their probability distributions matched our expectations: they become more step-like and approach a limiting distribution asymptotically as the total amount of consensus signaling increases. The pace of the sharpening of these curves depends on various model components, including the cell-cell coupling strength λ and the pulse period τ. We have added a new figure detailing these simulations (Fig. S5, included below), and we discuss this point in the text.

We also note that this increase in response fidelity towards a step-like function with increasing signal has been observed experimentally. It has been shown that increases in paracrine signaling during wound healing among a cell population can increase the response fidelity, which accounts for the sharpening of the fate curve [6].

© 2021. Published by The Company of Biologists under the terms of the Creative Commons Attribution License (https://creativecommons.org/licenses/by/4.0/).

Supplementary Figure 5

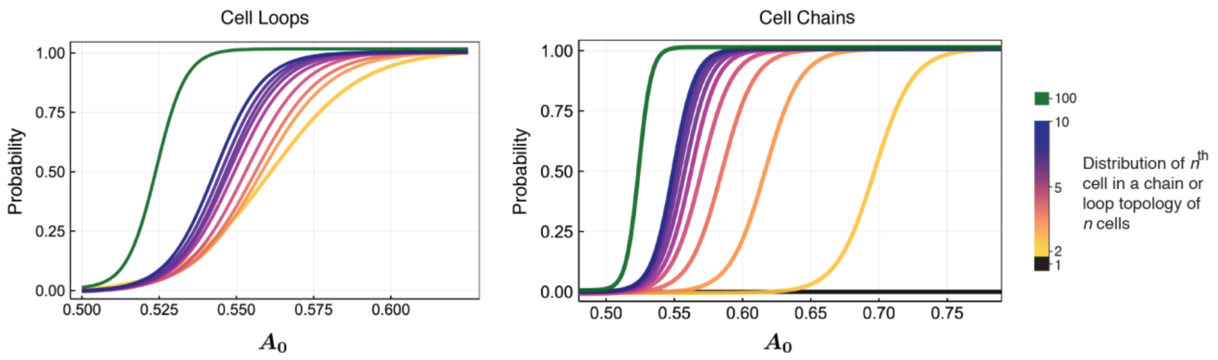

Comment 3: The authors carefully considered various topologies as in Figure 3. However, I wonder which of them are realistic and how can one extend those to the actual hematopoiesis system. I regard hematopoiesis as a well mixing system rather than having a fixed topology between cells. Also, I believe the spatial component would be important. Do you think if we consider something like a three dimensional agent based model, similar conclusion would hold?

Response: These are all important considerations, and the reviewer raises an excellent point regarding spatial considerations. Our model has no explicit spatial dimension, but we can consider space in the context of a cell's "connectedness" (highly connected cells being in closer proximity in space). To explore this, we simulated two new topologies which idealize cellular signaling behaviors in vivo and demonstrate how cell fate is linked to spatial organization. The first of these is a fully connected topology, representing cells that do not occupy spatially restricted niches: they are proximal or in a well-mixed environment. The second topology is a bidirectional cell chain, representing a spatially structured model where cells can only interact with their nearest neighbors.

The differences in cell fate commitment under these topologies was striking: whereas well- mixed cells are equally dependent on the fate choices of all other cells (as we would expect), in the case of the bidirectional cascade, cell proximity strongly influenced fate commitment (see new Fig. 6 in the paper). We expect signaling in the hematopoietic niche to lie between the extremes of these two topologies; the exact specification of cell-cell signaling topologies in vivo or in vitro requires data which is beyond current technologies, however the predictions this model makes regarding the influence of space on cell fate could be directly tested in vitro or in vivo.

We discuss these results in detail in the revised manuscript, in a new results section that we have added: "Spatial structure in communication networks impacts cell fate outcomes". We have also added a figure (Fig. 6).

With regards to agent-based modeling, indeed such an approach has certain clear advantages for studying spatial configurations of cells. However, our current approach relies on (and benefits from) the analytical tractability of the steady states and bifurcation behavior of the ODE system. Adapting this into an ABM framework will be at the expense of this tractability, and thus alternative methods will be required. This is outside the scope of the current work; we have added this point to the Discussion.

Comment 4: Can you provide some biological examples of consensus signaling and inhibition signaling of ME and GM? I think it would help biologist to further design experiments and measure those signals.

Response: We are grateful to the reviewer for highlighting this discussion point, which was lacking in the previous version. In the revised manuscript we discuss examples of consensus and dissensus signaling. We choose examples that 1) impact hematopoiesis, 2) are likely to be active in the bone marrow environment, and 3) are thus relevant to the determination of megakaryocyte/erythrocyte and granulocyte/monocyte cell fates. A canonical example of a dissensus signal, both in the context of hematopoiesis and other essential cell processes, is the Delta-Notch signaling pathway.

© 2021. Published by The Company of Biologists under the terms of the Creative Commons Attribution License (https://creativecommons.org/licenses/by/4.0/).

There are many examples of positive feedback in cellular signaling pathways that lead to consensus signaling. These occur both in healthy niches and in cancer (where positive feedback, e.g. mediated via Wnt/beta-catenin often influences unbounded cell growth). In the case of healthy niches, the cytokine TNF-α activates the p38 MAPK pathway, a pathway known to be differential between GM and ME cells [7].

Comment 5: I understand that for simplicity, only A is modified to incorporate external signaling, but I wonder what effects will considering noise in all B and C have. Will it add more stochasticity or cancel each other to stabilize the noise effect?

Response: This is an interesting question to explore. In vivo, multiple noisy signals are in competition. To test the effect of multiple signals, we developed two new models: one with signaling only via the parameter B, and one dual signaling model with signaling mediated by consensus signals in A and B simultaneously. (We omitted exploration of the parameter C because it affects X, which is a cofactor in the model rather than one of the master fate regulator genes.) In the dual-signaling model, we define consensus signaling with respect to B similarly to the original one-signal model:

$$B_2(t) = \begin{cases} \frac{P_1(t_s)+\lambda_B}{G_1(t_s)} B_2(t_s), & \text{for } \; t \in [t_s, t_s+\tau) \text{ and } \lambda_B > 0 \\ B_0, & \text{otherwise} \end{cases}$$

We tested this model in both chain and loop topologies. We also tested these topologies where there were consensus signals changing both parameters A and B. We found a bistable region in the joint parameter space of (A0, B0), and representative simulations are plotted in a new figure (Fig. S7, shown below). These simulations demonstrate how the combination of signals in A and B jointly impact cell fate probability distributions. Though we note that, due to the complexity of the two-signal parameter space, we have not fully explored its behaviors. In the example shown for a two-cell chain, joint signaling sharpens the fate distribution (Fig. S7A). In the two-cell loop, joint signaling sharpens to distribution to a lesser extent but shifts the point of cell fate commitment to lower B values. We have added a discussion of these interesting competing- signal results to the main text.

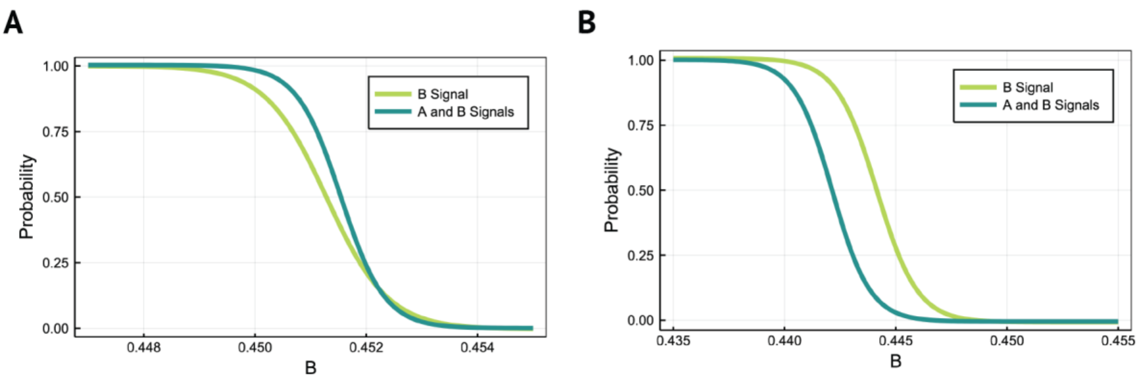

Figure S7: Probability distributions of (A) cell two in a chain and (B) two cell loop converging to the G highs state resulting from a consensus signal to the parameter B and dual consensus signals to both parameters A and B. A_0 is fixed as 0.65. The signaling parameters for the signals changing A and B are λ _A = 28.0 and λ _B = 28.0 respectively.

Comment 6: In the subsection on page 8, when you discuss the importance of parameter λ, will altering κ have similar effects? Can you comment on that?

Response: We recognize the need for greater investigation into/discussion of κ. In the revision we have thoroughly investigated its effects on simple signaling topologies. In doing so, we realized that for consistency, the dissensus signal should be defined such that κ satisfies the following inequalities:

© 2021. Published by The Company of Biologists under the terms of the Creative Commons Attribution License (https://creativecommons.org/licenses/by/4.0/).

$$\frac{\kappa + P_{\{\min\ P \text{ High State}\}}}{G_{\{\max\ P \text{ High State}\}}} > 1, \qquad \frac{\kappa + P_{\{\max\ G \text{ High State}\}}}{G_{\{\min\ G \text{ High State}\}}} < 1, \qquad \kappa > 0.$$

These inequalities are satisfied for κ ∈ [15.495, 39.997], i.e. the same parameter range as for λ. Our initial reasoning for placing the parameters λ and κ in the denominator of the signaling term was to avoid unrealistic behaviors when the concentrations of G or P were near zero. Thus, for consistency, we leave Fig. 3 unchanged. In a new supplemental figure, we explore the effects of dissensus signaling on the distribution of the two-cell chain by varying κ (analogous to analysis of the consensus signal in Fig. 4A. Unlike in Fig 4, we did not observe coordination between cell fates as we vary κ, resulting from the fact that the dissensus signal drives communicating cells towards opposite fates.

We have added these updates to the Methods:
Similarly for κ, in order to satisfy the analogous inequalities for the dissensus signal, we redefine the dissensus signal as

$$A_2(t) = \begin{cases} \frac{P_1(t_s)+\kappa}{G_1(t_s)} A_2(t_s), & \text{for } t \in [t_s, t_s+\tau) \text{ and } \kappa > 0 \\ A_0, & \text{otherwise} \end{cases}$$

where κ ∈ [15.495, 39.997].

We have also added the following supplementary figure:

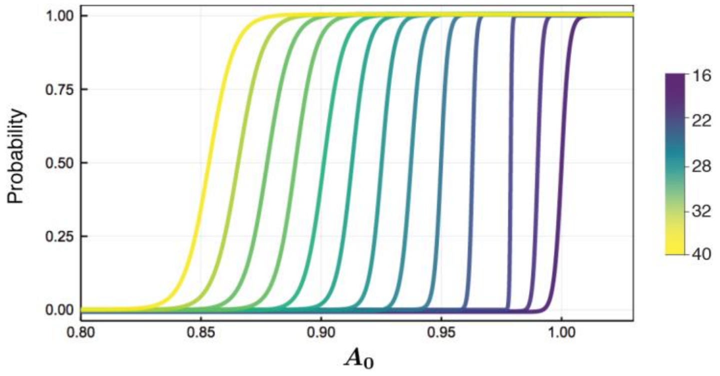

Figure S8: Probability distributions of cell fate commitment to G high steady state for cell 2 in a chain of two cells with a dissensus signal where κ ∈ [16, 40].

Comment 7: I wonder if the results without the extrinsic noise in the signaling is significant enough. In Figure 3, I see the probability becoming closer to a step function as the number of cell n increases which would be closer to reality, and in Figure 4, the interesting A0 seems to be a very tuned value that happens by changing A0 from 1 to 1.002, which is 0.2% of the base value.

Response: The reviewer is quite correct to point out that in the absence of extrinsic noise, the range of uncertain fates in A0 can be less than 1% of the base value. The size of this range is mainly due to the initial conditions chosen: we assume that all cells are homogeneous thus start with the same initial conditions in Gata-1 and PU.1. This is an artificial scenario that we imposed as our goal was to investigate other sources of variability (e.g. extrinsic/intrinsic noise) while controlling for cell heterogeneity. In vivo, there will almost certainly be other sources of variability, and these will lead to significantly more variable (wider) cell fate choice probability distributions.

We also note that the uncertain region of fate probability is closer to 10% when the total number of signals received is smaller. It is not currently possible to know how many signals a multipotent hematopoietic cell in the bone marrow receives. Over the fairly narrow temporal window of cell fate commitment, we reason that it could be a relatively small number of signals, in the range of 5-10 rather than hundreds, which in our model would result in a variable fate region of around 10% of the base value.

© 2021. Published by The Company of Biologists under the terms of the Creative Commons Attribution License (https://creativecommons.org/licenses/by/4.0/).

Minor comments:

- In the end of page 6, after 'decrease wait time to μ = 40', can you elaborate on 'order to distinguish between the effects'?

Response: We added the following to clarify this point:

"In the analysis of noise effects below, we decrease the mean wait time to μ = 40, resulting in a larger signal to wait time ratio, allowing signals to have a greater influence on target cells. Doing so accentuates the effects of intrinsic and extrinsic noise because cells experience more signals in the pulsed state relative to the baseline."

- In Figure 5B, if you plot the lines with the same x axis scale as in C and D, I think it would be more straightforward to compare, but I will the decision to you.

Response: We appreciate the suggestion and assessed this, but plotting over the wider x range obscures the finer details of the curve features in B, thus we leave the original scale.

- In page 7, testing 20 cell loop topology, can you refer to Figure 3 where you have the figure of cell topology?

Response: We have added this reference where we introduce the 20-cell loop results.

- In page 4, why don't you merge the discussion of introducing λ, κ values as one (λ = κ = 1) and explaining how to choose the range as you did in page 5.

Response: This is a great suggestion. In the revision we thoroughly revised our introduction of both λ and κ --- in doing so they are now introduced together.

References cited in this letter:

1. **Nadler, B., Naeh, T. and Schuss, Z.** (2001). The stationary arrival process of independent diffusers from a continuum to an absorbing boundary is poissonian. *SIAM Journal on Applied Mathematics* **62**, 433-447.
2. **Hérault, A., Binnewies, M., Leong, S., Calero-Nieto, F. J., Zhang, S. Y., Kang, Y.-A., Wang, X., Pietras, E. M., Chu, S. H., Barry-Holson, K., Armstrong, S., Göttgens, B. and Passegué, E.** (2017). Myeloid progenitor cluster formation drives emergency and leukaemic myelopoiesis. *Nature* **544**, 53-58.
3. **Giladi, A., Cohen, M., Medaglia, C., Baran, Y., Li, B., Zada, M., Bost, P., Blecher- Gonen, R., Salame, T.-M., Mayer, J. U., David, E., Ronchese, F., Tanay, A. and Amit, I.** (2020). Dissecting cellular crosstalk by sequencing physically interacting cells. *Nature Biotechnology* **38**, 629-637.
4. **Brewer, C., Chu, E., Chin, M. and Lu, R.** (2016). Transplantation dose alters the differentiation program of hematopoietic stem cells. *Cell Reports* **15**, 1848-1857.
5. **Müller, E., Grinenko, T., Pompe, T., Waskow, C. and Werner, C.** (2015). Space constraints govern fate of hematopoietic stem and progenitor cells in vitro. *Biomaterials* **53**, 709-715.
6. **Handly, L., Pilko, A. and Wollman, R.** (2015). Paracrine communication maximizes cellular response fidelity in wound signaling. *eLife* **4**, e09652.
7. **Wang, J., Tu, C., Zhang, H., Huo, Y., Menu, E. and Liu, J.** (2021). Single-cell analysis at the protein level delineates intracellular signaling dynamic during hematopoiesis. *BMC Biology* **19**, 201.

© 2021. Published by The Company of Biologists under the terms of the Creative Commons Attribution License (https://creativecommons.org/licenses/by/4.0/).

Second decision letter

MS ID#: DEVELOP/2021/199779

MS TITLE: A single-cell resolved cell-cell communication model explains lineage commitment in hematopoiesis

AUTHORS: Megan K Rommelfanger and Adam L MacLean
ARTICLE TYPE: Research Article

I am happy to tell you that your manuscript has been accepted for publication in Development, pending our standard ethics checks.

Reviewer 1

Advance summary and potential significance to field

(same as last revision)

Comments for the author

The authors have thoroughly addressed all the questions/comments I had in my previous review. I would recommend the publish of the manuscript in Development

Reviewer 2

Advance summary and potential significance to field

Summarized in my first review.

Comments for the author

The authors answered all my comments. I recommend this manuscript for publication.

© 2021. Published by The Company of Biologists under the terms of the Creative Commons Attribution License (https://creativecommons.org/licenses/by/4.0/).
